# Assessing the damage: analyzing the impact of the COVID-19 pandemic on accelerometer-assessed 24-hour movement behaviours in Brazilian adolescents

**DOI:** 10.1186/s12889-025-23155-8

**Published:** 2025-05-31

**Authors:** Marcus V. V. Lopes, Ian Janssen, Bruno G. G. da Costa, Bruno N. de Oliveira, Gabrielli T. de Mello, Jean-Philippe Chaput, Kelly S. Silva

**Affiliations:** 1 Healthy Active Living and Obesity Research Group, Children’s Hospital of Eastern Ontario Research Institute, 401 Smyth Rd, Ottawa, ON K1H 8L1 Canada; 2 School of Kinesiology and Health Studies, Queen’s University, Kingston, ON Canada; 3 Department of Public Health Sciences, Queen’s University, Kingston, ON Canada; 4 Department of Kinesiology and Physical Education, McGill University, Montréal, QC Canada; 5 Department of Physical Education, Federal University of Santa Catarina, Florianópolis, SC Brazil; 6 Institute for Advancing Health Through Agriculture, Texas A&M AgriLife Research, Dallas, TX USA

**Keywords:** Motor Activity; Sleep, Accelerometry, Movement Sensor, Compositional Data

## Abstract

**Background:**

Although there is consistent evidence of unhealthy changes in the 24-h movement behaviours when comparing pre-COVID-19 periods to the early stages of the pandemic, there is limited research on long-term changes among adolescents. This study aimed to analyze both between- and within-participant differences in accelerometer-assessed 24-h movement behaviours by comparing cross-sectional and prospective data from the pre-COVID-19 period (August to December 2019) to the period following the reopening of schools for in-person classes in southern Brazil (August to December 2022).

**Methods:**

This is a repeated cross-sectional design with a nested cohort. The 24-h movement behaviours (i.e., light physical activity [LIPA] and moderate-to-vigorous physical activity [MVPA], sedentary behaviour [SB], and sleep time [SPT]) were assessed by processing raw accelerometer data derived from a 24-h/7-day wrist-worn protocol. Compositional multilevel models were applied to compare the 24-h movement behaviour composition between time points for both cross-sectional and prospective data. Self-reported sociodemographic characteristics were examined as potential moderators.

**Results:**

The cross-sectional and prospective samples comprised, respectively, 1276 (53% female, average age of 16.4 ± 1.1) and 249 (53% female, average age of 15.6 ± 0.8) participants. The 24-h movement behaviour composition differed between time-points in the cross-sectional (*p* < 0.001) and prospective samples (*p* < 0.001). Differences from 2019 to 2022 were explained by lower MVPA (-3.3 and -5.4 min/day in cross-sectional and prospective analysis, respectively) and a higher SB (4.7 and 34 min/day in cross-sectional and prospective analysis, respectively). No significant differences were observed for LIPA and SPT.

**Conclusions:**

Differences in the 24-h movement behaviour composition comparing the cross-sectional samples, although statistically significant, were considered trivial and unlikely to have a substantial practical impact. However, considerable differences were observed in the prospective analysis. The results suggest that most of the observed changes over time were expected as a natural consequence of aging during high school, with only a small portion attributable to the residual impact of the pandemic.

**Supplementary Information:**

The online version contains supplementary material available at 10.1186/s12889-025-23155-8.

## Introduction

The outbreak of the novel coronavirus, SARS-CoV-2, and the subsequent declaration of the COVID-19 pandemic in early 2020 [1], led to the implementation of global efforts to mitigate community transmission. The primary preventive measures, including social distancing and isolation policies were effective in delaying the spread of the disease and preventing uncontrolled infection rates. However, these measures required abrupt social and structural changes that significantly disrupted the routines of school-aged adolescents [2]. This population, who spends a substantial proportion of their waking hours at school, was directly affected by the closure of community areas (e.g., schools, gyms, clubs) and the transition from in-person to remote school classes. A public health concern that emerged was related with the impact of social distancing policies on how adolescents use their time. From a movement perspective, adolescents should pursue an optimal balance of using their time by increasing the time spent in physical activity at both light (LIPA) and moderate-to-vigorous intensity (MVPA) while limiting the time spent in sedentary behaviours (SB) and maintaining adequate sleep time (SPT) [3, 4]. This set of behaviors comprise the construct of the 24-h movement behaviours, which describes the pattern of body movement (below or above resting-equivalent energy expenditure) that occurs when individuals are awake or asleep and is constrained to 24 h per day [5, 6]. However, the implementation of rigorous restrictive measures accompanied by strong stay-home messages and changes in youth routines during the early stages of the COVID-19 pandemic did not favor healthy time-use patterns.

Evidence from systematic reviews show that the 24-h movement behaviours were negatively affected during the first year of the pandemic [7–10]. A systematic review using self-reported and accelerometer-measured data show that adolescents reduced their time spent in MVPA by 28 min/day while no changes were confirmed for LIPA compared to the pre-pandemic period [7]. Sedentary time considerably increased with consistent evidences from both cross-sectional and prospective studies [9, 10]. Such behavioural changes, however, appear to vary based on the stringency of restrictive measures, which became more relaxed after the first waves of the pandemic. For instance, studies comparing periods of strict lockdown to phases of school reopening or policy relaxation observed increased physical activity levels in adolescents [11, 12], which may suggest a partial or full recovery from the declines seen during lockdown. However, findings were not consistent, as exemplified by a prospective study where stable accelerometer-assessed MVPA alongside increased SPT were observed among Swedish adolescents [13]. Considering that the easing of COVID-19 related restrictions occurred at different time points between countries and territories, the evidence from post-restrictions, particularly the opening of schools, is scarce.

Although the literature on changes in 24-h movement behaviours during the early stages of the COVID-19 pandemic is well documented, the following limitations characterize the current state of evidence [8, 9]: (a) most of the available evidence accounted for comparisons within the first year of the pandemic; (b) most studies were of cross-sectional design and used self-reported measures of the 24-h movement behaviours; (c) most studies analyzed the movement behaviours individually (i.e., not accounting for the 24-h time-use framework as recommended [14] to account for the compositional structure of the data; and (d) most samples were from high-income countries, and with considerable variation between effect-sizes. Evidence from countries with pronounced health inequities, such as Brazil, is lacking. The response of the Brazilian government to the pandemic included a slow rollout of vaccination coverage, a collapse of the healthcare system [15], and a denialist political leadership [16]. As a result, the country had a mortality rate four times the global average, contributing to 11% of global COVID-19 deaths by the pandemic's end [15].

Thus, the present study, conducted with high-school-aged students in Southern Brazil, applied a compositional data approach to (1) analyze between- and within-participant differences in accelerometer-assessed 24-h movement behaviours by comparing cross-sectional and prospective data between the periods prior to COVID-19 and after the reopening of schools for in-person classes; and (b) examine whether these differences varied according to sociodemographic characteristics.

## Methods

### Study design and participants

This study utilized a repeated cross-sectional design with a nested cohort, analyzing cross-sectional and prospective data from the ELEVA study (Portuguese acronym translated as Longitudinal Study of the Lifestyle of Adolescents). The ELEVA study examined health indicators and lifestyle of high-school students from the metropolitan region of Florianópolis, southern Brazil, in 2019 and 2022. In both survey years, all three public schools in the region that offered high-school diplomas integrated with professional courses accepted to be part of the study. These courses are characterized by having three years of traditional high school course work followed by one year devoted to technical training courses.

All students in the first three years of high school were eligible for the repeated cross-sectional sample in 2019 and 2022. Those in the first year in 2019 were eligible for the nested cohort as they were expected to be completing high school between the end of 2022 and early 2023. A census method was adopted, and all students attending classes during the data collection periods were invited to participate except those who were unable to take part in the study measurements due to injury, illness, or disabilities. Out of 2,320 students eligible for the repeated cross-sectional sample, 2,008 agreed to participate and were included in the study. A total of 333 participants who were assessed in 2019 were registered as students in the same institutions in 2022 and were eligible for the longitudinal sample. Flow charts detailing the enrollment of the participants are provided in Figures S1-S2 (Additional File 1). The data collection was performed over three visits to each class at the schools. In both study years, the data collection occurred from August to December, which corresponded to the second semester of the school year. The period of assessment for each school was similar in both survey years to reduce biases related to seasonality (e.g., a school assessed during early summer in 2019 was also assessed in 2022 early summer). The data collection period in 2022 occurred the first semester after the transition from remote learning to full in-person classes at the school (officially declared at June 6, 2022) [17].

### Measures

#### 24-hour movement behaviours

The 24-h movement behaviours (i.e., MVPA, LIPA, SB and SPT) were assessed using triaxial Actigraph GT3x + and wGT3x + accelerometers (ActiGraph Corporation, Pensacola, Florida, USA). Participants were instructed to wear the accelerometer on their non-dominant wrist for 24 h on seven consecutive days. Trained research staff attached the monitors to the participant’s wrist using a disposable PVC band and instructed them to remove the device only during activities involving full submersion in water (e.g., swimming, surfing) but not during other water-based activities (e.g., showering, washing dishes). Extra disposable bands were made available. This protocol was slightly adapted based on previous analyses showing that instructions for removal for any water-based activities may negatively impact compliance with wear-time criteria in 24-h protocols [18]. Accelerometer non-wear time based on triaxial data was assessed using a validated algorithm [19]. Participants who provided data for at least 16 h/day over a minimum of three weekdays and one weekend were included in the analyses. Although the criteria of 16 h/day was used as traditionally applied for measuring the 24-h behaviours, the average wear time among participants who met the wear criteria was 23.8 h.

Accelerometer raw data were collected at a frequency of 30 Hz, calibrated to local gravity, and expressed as Euclidean norm minus one (ENMO) using 5-s epochs. Time gaps identified as non-wear time through each 24-h interval that reached the validation criteria (≥ 16 h) were imputed at the raw-data level based on average values across days. Thus, all valid days are normalized to 24 h. Each epoch in a 24-h window was classified as SB (< 35.6 mg), LIPA (≥ 35.6 and < 201.4 mg) or MVPA (≥ 201.4 mg) using the Hildebrand cut-off points [20, 21] recommended for adolescents [22]. The SPT was obtained using the Heuristic algorithm that examined the distribution of change in Z-angle. This algorithm differentiates sleep from other inactivity windows by calculating the longest sustained period of inactivity with the lowest number of interruptions in a 24-h time window [23]. The parameter sleep window, which is commonly referred as a proxy measure of time in bed, was used as SPT. The time spent in MVPA, LIPA, SB, and SPT were weighted according to weekdays and weekends (5:2 ratio) and averaged across days for each participant using a midnight-to-midnight day definition. The accelerometer data were processed using the “GGIR” package version 2.9.1 [24] in R software, version 4.3.0 (R Foundation for Statistical Computing, Vienna, Austria).

The 24-h movement behaviours were treated as a compositional variable. As the accelerometer variables were aggregated based on a midnight-to-midnight day definition, the composition is constrained to 1440 min (i.e., 24 h). To account for the properties of compositional data, the set of variables was transformed into three isometric log-ratio (ILR) coordinates using a sequential binary partition [6]. Compositional data were treated in the R packages *Compositions* and *robCompositions*.

#### Sociodemographic factors

Participants answered a self-reported online questionnaire on SurveyMonkey® using their smartphones during school classes. The average completion time was 24 min. The following sociodemographic characteristics were analysed: sex (male or female); age (years); highest education of a parent/legal guardian (< 8 years, 9–11 years, > 11 years or don’t know); and family structure (live with both parents, live with a single parent, does not live with parents). Socioeconomic status (SES) was obtained by a scoring system proposed by the Brazilian Association of Research Companies to represent a countrywide standardized measure of SES in Brazil [25]. The scoring is applied to household belongings (i.e., number of bathrooms, housemaids, cars, computers, dishwashers, fridges, freezers, washing machines, DVD players, microwave ovens, motorcycles, drying machines; highest education level of the family; having piped water, living in a paved street) reported by participants. Each item was weighted and summed into a score ranging from zero to 100, with higher values indicating higher family wealth.

### Statistical analyses

#### Descriptive statistics and retention analysis

Descriptive statistics were used to characterize the cross-sectional and longitudinal samples. Means and standard deviations (SD) were used to describe continuous variables while absolute and relative frequencies was used for nominal variables. The geometric mean (i.e., the relative contribution of each behaviour to the 24 h expressed as proportions) and the variation matrix (i.e., the magnitude of co-dependency between components of the composition with lower values expressing higher correlations) were used to describe the 24-h movement behaviour composition.

Sociodemographic characteristics were compared between the following groups: (a) repeated cross-sectional samples, (b) participants included and excluded due to no-valid accelerometer data for all samples, (c) nested longitudinal sample and the first wave of the cross-sectional sample (i.e., 2019), (d) participants assessed at both time points and dropouts in the longitudinal sample. Pearson x^2^ and Wilcoxon rank sum tests were used to compare numerical and categorical variables, respectively.

#### Main analysis

All analyses modeled the 24-h movement behaviours as a compositional dependent variable, allowing examination of whether the relative distribution of time spent in each behaviour (i.e., MVPA, LIPA, SB, and SPT) varied as a function of a set of independent variables. Consequently, any change in the composition reflects a reallocation of time among behaviours – an increase in one component necessarily implies a decrease in one or more of the other behaviours. To select an appropriate modeling strategy, the following considerations were taken into account: (a) the analysis needed to accommodate multivariate outcomes (i.e., correlated dependent variables, the ILR coordinates, modeled jointly); and (b) the analysis had to account for the hierarchical structure of the data (i.e., repeated measures nested within individuals, who were in turn nested within schools). Accordingly, multilevel compositional models for stacked data were used. This approach, recently introduced by von Rosen and colleagues [26], has been applied to repeated-measures data [27]. All analyses were performed in R, and the code, which includes a step-by-step explanation of the procedures, is available in the Additional File 2.

The stacked regression approach requires reshaping the dataset to a longer format and stacking the values of ILR coordinates for each participant, which are identified by a three-level factor variable. Thus, the cross-sectional dataset includes three observations per individual, one for each ILR coordinate, while the longitudinal dataset includes six observations per individual, one for each ILR coordinate at each time point (i.e., 2019 and 2022). The values of the ILR coordinates were then regressed on the interaction between the ILR factor variable and time point. Thus, a type-III likelihood test was performed to determine whether the composition differed between time points (ILR factor by time interaction term). The time variable refers to between-individual differences in the cross-sectional analysis and to within-individual changes over time in the prospective analysis.

Multilevel mixed models were used to analyze composition differences between survey waves in the repeated cross-sectional samples and changes over time in the longitudinal sample accounting for clustering at the school level. Cross-sectional data were modelled using a two-level linear model for a Gaussian distribution. The model included random intercepts for ILR coordinates values within individuals and for individuals nested within schools. The variables age, sex, SES, and family structure and their interaction with time were included as covariates. Prospective data were modelled using a similar model specification but with time invariant covariates (i.e., characteristics at 2019), and the inclusion of a random slope for the ILR factor at the individual level to account for the repeated measures of compositions over time and the correlation between random intercepts and slopes. Given that mixed models can handle unbalanced data, dropouts from the prospective data (missing data in 2022 but not in 2019) were retained in the analyses. Models were used to extract the predicted ILR coordinates which were back-transformed into geometric averages per time point.

A second set of analysis was performed using a pivot partition to create the ILR coordinates and examine which composition components differed between time points. The first pivot coordinate contained information on the contribution of a single behaviour (e.g., MVPA) relative to the geometric average of the remaining composition (e.g., LIPA, SB and SPT). In the context of the stacked data, the main slope of time refers to its effect on the first pivot coordinate. Thus, four sets of pivot coordinates were created by rotating the components of the composition and were modelled using the same approach.

A third set of analyses were performed to examine whether age, sex, SES, or family structure modified the time differences in the 24-h movement behaviours. Associations were tested by including the interaction term of each covariate to the “ilr factor by time” slope (e.g., ilr factor * time * sex). In the case of significant interaction terms (p < 0.05), subgroup effects were examined by performing a linear combination of coefficients.

#### Sensitivity analysis

All the analyses were repeated accounting for: (a) the exclusion of compositional outliers detected based on the inspection of Mahalanobis Distance; and (b) complete case analysis for the prospective data (i.e., retaining only participants who provided valid data at both 2019 and 2022 time points).

Although the cut-off points used to estimate MVPA, LIPA, and SB were used in previous studies with adolescents and are currently recommended for this age group [22], they were developed in a sample of children (7–11 years) and, currently, there are no published cut-off points developed specifically for the age of participants in this study. Thus, all the main analysis were replicated using accelerometer estimates of SB (< 44.8 mg), LIPA (≥ 44.8 and < 100.6 mg) and MVPA (≥ 100.6 mg) derived from adults (18–65 years) cut-off points [20, 21].

## Results

### Cross-sectional data

Out of 2,008 participants included in the repeated cross-sectional sample, 666 and 599 provided valid data for all variables of interest in 2019 and 2022, respectively. Reasons for exclusion (e.g., incomplete questionnaire, non-valid accelerometer data) are described in Figure S1 (Additional File 1). Comparisons between participants who provided valid accelerometer data and those excluded for not complying with the accelerometer protocol in 2019 (n = 124) and 2022 (n = 236) are presented in Tables S1-S2 (Additional File 1), respectively. Compared to those excluded from the 2022 analysis due to non-compliance, included participants had a lower average SES score (38.7 versus 41.2) and a smaller proportion were males (44% versus 53%). None of the remaining sociodemographic characteristics differed according to accelerometer compliance in 2019 or 2022.

Participants (53% female, average age of 16.4 ± 1.1) were mostly living with both parents (61%), and had parents with a high education level (59% with > 11 years of study). Participants’ characteristics according to the wave of study are presented in Table 1. No significant differences were observed for sociodemographic characteristics between waves, except for age, which was 0.2 years lower, on average, among participants of the 2019 wave.

**Table 1:** Participant’s characteristics in repeated cross-sectional samples

Variable	Total sample *N* = 1,276	2019 sample *N* = 679	2022 sample *N* = 597	*p*-value*
Sex, n (%)				0.059
Male	598 (47%)	335 (49%)	263 (44%)	
Female	678 (53%)	344 (51%)	334 (56%)	
Age (years), mean (SD)	16.4 (1.1)	16.3 (1.1)	16.5 (1.2)	0.019
Highest education among parents, n (%)				0.100
< 8 years	73 (5.7%)	40 (5.9%)	33 (5.5%)	
8–11 years	405 (32%)	227 (33%)	178 (30%)	
> 11 years	759 (59%)	398 (59%)	361 (60%)	
Do not know	39 (3.1%)	14 (2.1%)	25 (4.2%)	
SES score (0—100), mean (SD)	38.9 (10.0)	39.1 (9.8)	38.7 (10.2)	0.427
Family structure, n (%)				0.136
Live with both parents	780 (61%)	431 (63%)	349 (58%)	
Single parent	430 (34%)	212 (31%)	218 (37%)	
Do not live with parents	66 (5.2%)	36 (5.3%)	30 (5.0%)	

*SD* Standard Deviation, *SES* Socioeconomic Status

^*^Wilcoxon rank sum test applied to age and SES, and Pearson x^2^ tests applied to the remaining variables

**Fig. 1:**
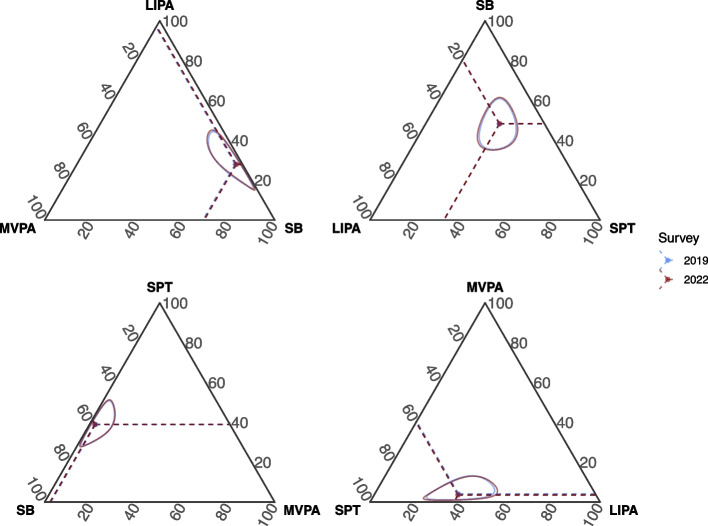
Ternary diagrams describing the 24-h movement behaviour composition among high school students from the repeated cross-sectional samples (2019 and 2022), Brazil. Solid-coloured circles and dashed lines represent the unadjusted compositional mean. Coloured areas refer to the ternary 95% confidence intervals. MVPA, moderate-to-vigorous-intensity physical activity; LIPA, light-intensity physical activity; SB, sedentary behaviour; SPT, sleep duration. The relative proportion of each behaviour to the 24 h was 2.1% MVPA, 19.2% LIPA, 47.2% SB and 31.4% SPT in 2019; and 1.9% MVPA, 19.2% LIPA, 48.5% SB and 31.5% SPT in 2022

The unadjusted compositional mean and ternary confidence intervals of the 24-h movement behaviours are presented in Fig. 1. The comparison of the adjusted time-use composition between waves is presented in Table 2. The 24-h movement behaviour composition differed between waves (*p* < 0.001), which was primarily explained by a slightly lower MVPA (−11%, equivalent to 3.3 min/day, *p* < 0.001), and a higher SB (0.7%, equivalent to 4.7 min/day, *p* = 0.013). The supplementary materials contain additional information on the compositional variation matrices and the models’ specifications (Additional File 2).

**Table 2:** Comparison of 24-h movement behaviour composition between 2019 and 2022 cross-sectional samples

Outcomes	Adjusted predictions (min/day)^a^		Difference (%)	*p*-value
	2019 sample	2022 sample		
Composition				< 0.001
MVPA	29.9	26.6	−11.0	< 0.001
LIPA	275.8	274.0	−0.7	0.120
SB	679.6	684.3	0.7	0.013
SPT	454.7	455.1	0.1	0.040

Models were adjusted for age, sex, SES, and family structure

*MVPA* moderate-to-vigorous-intensity physical activity, *LIPA* light-intensity physical activity, *SB* sedentary time, *SPT* sleep time

^a^Marginal means adjusted for mean age and proportionally weighted according to the levels of factor covariates

### Longitudinal data

Of the 333 participants of the 2019 wave enrolled in the schools in 2022, 249 provided complete data in 2019 and were analyzed. Reasons for losses are described in Figure S2 (Additional File 1). Losses due to non-compliance with the accelerometer protocol were not associated with sociodemographic characteristics (Table S3, Additional File 1). Out of the 249 participants, 138 provided valid data in 2022 (retention rate of 55%). Most losses to follow-up reflected school dropouts (65 of 111) as these students were not attending classes during the data collection period. Dropouts were slightly older than those who were assessed at both time points (Table 3).

**Table 3:** Participant’s characteristics in the longitudinal sample

Variable	Time 1 (2019) *N* = 249	Time 2 (2022) *N* = 138	Dropouts *N* = 111	*p*-value*
Sex, n (%)				0.829
Male	117 (47%)	64 (46%)	53 (48%)	
Female	132 (53%)	74 (54%)	58 (52%)	
Age (years), mean (SD)	15.6 (0.8)	15.4 (0.7)	15.8 (0.9)	< 0.001
Highest education among parents, n (%)				0.406
< 8 years	12 (4.8%)	6 (4.3%)	6 (5.4%)	
8–11 years	93 (37%)	46 (33%)	47 (42%)	
> 11 years	137 (55%)	81 (59%)	56 (50%)	
Do not know	7 (2.8%)	5 (3.6%)	2 (1.8%)	
SES score (0—100), mean (SD)				0.871
Family structure, n (%)	38.5 (9.4)	38.6 (9.8)	38.4 (9.0)	
Live with both parents				0.870
Single parent	159 (64%)	87 (63%)	72 (65%)	
Do not live with parents	80 (32%)	46 (33%)	34 (31%)	

*SD* Standard deviation, *SES* Socioeconomic status

^*^Wilcoxon rank sum test applied to age and SES, and Pearson x^2^ tests applied to the remaining variables

**Fig. 2:**
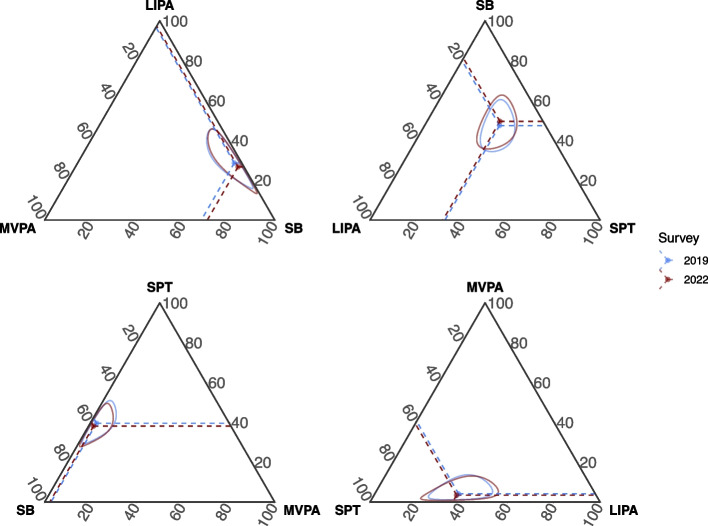
Ternary diagrams describing the 24-h movement behaviour composition among high school students from the longitudinal sample in 2019 and 2022, Brazil. Note: Solid-coloured circles and dashed lines represent the unadjusted compositional average. Coloured areas refer to the ternary 95% confidence intervals. MVPA, moderate-to-vigorous-intensity physical activity; LIPA, light-intensity physical activity; SB, sedentary time; SPT, sleep duration. The relative proportion of each behaviour to the 24 h was 2.2% MVPA, 19.5% LIPA, 46.4% SB and 31.9% SPT in 2019; and 1.8% MVPA, 18.4% LIPA, 48.7% SB and 31.1% SPT in 2022

The unadjusted compositional mean and ternary confidence intervals of the 24-h movement behaviours in the longitudinal sample in 2019 and 2022 are presented in Fig. 2. The within-participant changes in the time-use composition are presented in Table 4. A change in the composition was observed from 2019 to 2022 (*p* < 0.001). The proportion of time allocated to SB relative to the remaining behaviours increased (5.1%, equivalent to 34 min/day, *p* < 0.001), while the contribution of MVPA to the remaining composition decreased from 2019 to 2022 (−17.7%, equivalent to 5.4 min/day, *p* < 0.001). Changes in the relative proportions of LIPA (−5.9%, equivalent to 16.3 min/day) and SPT (−2.6%, equivalent to 12.2 min/day) were not statistically significant – therefore, their contribution to the overall time reallocation is inconclusive.

**Table 4:** Within-participant changes in 24-h movement behaviour composition between 2019 and 2022 samples

**Outcomes**	**Adjusted predictions (min/day)**^a^		**Difference (%)**	*p*-value
	**2019**	**2022**		
Composition				< 0.001
MVPA	30.5	25.1	−17.7	< 0.001
LIPA	277.4	261.1	−5.9	0.923
SB	671.5	705.5	5.1	< 0.001
SPT	460.6	448.4	−2.6	0.084

Models were adjusted for sociodemographic characteristics at 2019 (i.e., age, sex, SES, and family structure)

*MVPA* moderate-to-vigorous-intensity physical activity, *LIPA* light-intensity physical activity, *SB* sedentary behaviour, *SPT* sleep time

^a^Marginal means adjusted for mean age and proportionally weighted according to the levels of factor covariates

The exploratory analyses on potential moderators of the time differences in the 24-h movement behaviours composition are presented in Table 5. None of the three-way interaction terms between the 24-movement composition, time (i.e., 2022 versus 2019), and sociodemographic variables were significant at *p*-value < 0.05. Thus, no subgroup analyses were performed.

**Table 5:** Moderation effects of sociodemographic factors on the comparisons of movement composition between 2019 and 2022

Interaction terms	df	Chi	*p*-value
Cross-sectional			
ILR * Survey * Sex	3	2.26095	0.520
ILR * Survey * Age	3	2.87403	0.411
ILR * Survey * Family structure	6	10.6766	0.099
ILR * Survey * SES	3	3.23735	0.356
Longitudinal			
ILR * Survey * Sex	3	2.39977	0.494
ILR * Survey * Age	3	3.33398	0.343
ILR * Survey * Family structure	6	5.5176	0.479
ILR * Survey * SES	3	4.39775	0.222

*ILR* Isometric log-ratio factor, *SES* Socioeconomic status

^*^Wald test

### Sensitivity analyses

The analyses accounting for the exclusion of compositional outliers and complete case filtering for the prospective sample let to the same conclusions (data not shown). The analyses performed using cut-off points designed for adults to classify SB, LIPA, and MVPA are available in Additional File 3. The 24-h movement behaviour compositions did not differ between 2019 and 2022 in the cross-sectional analysis (p = 0.061). Prospective changes were observed to be in the same direction but with a lower magnitude compared to the findings that were derived using the cut-off points for children.

## Discussion

This study compared the 24-h movement behaviour compositions among high-school students in the metropolitan region of Florianópolis, southern Brazil, focusing on behavioural changes from the period before the COVID-19 pandemic (2019) and after the reopening of schools for in-person classes (2022). The sample comprised students attending a high-school integrated with vocational courses from all three public high schools in the region – a population that is often underrepresented in national health surveys. The behaviours were assessed using accelerometers and were analyzed using compositional data analyses within a study featuring a cohort nested within a repeated cross-sectional survey. The adopted design is robust for investigating the effects of a pandemic that impacted the whole world, eliminating the possibility of having control groups. The nested cohort and a repeated cross-sectional sample allowed us to gain insights into how the observed behavioural changes differ from what is typically expected with aging during the high school years.

The analysis of repeated cross-sectional data revealed slight differences in the composition of 24-h movement behaviours when comparing students who did not face the COVID-19 pandemic before starting high school versus those high school students who returned to in-person instruction after COVID-19 restrictions were lifted. Although statistically significant, the differences (mostly explained by lower MVPA [3.3 min/day] and higher SB [4.7 min/day] in 2022 than in 2019) were quite small and are unlikely to have a meaningful impact on the overall health of the population. Although there is consistent evidence of lower MVPA [7] and increased SB [8, 9] and SPT [9, 10] when comparing the pre-COVID period with the earlier stages of the pandemic, studies have shown a trend toward diminishing differences in movement behaviour levels when comparisons are made with periods of relaxed restrictions. A prospective comparison of Swedish students that compared the pre-COVID-19 and mid-2021 periods, when schools remained closed in Sweden, found a decrease in SPT and LIPA, an increase in SB, and no change in MVPA [13]. Studies of adolescents that compared movement behaviours during COVID-19 with the behaviours levels after the return to school [11] and with the complete removal of social restrictions [12] observed higher levels of physical activity at the later periods. Collectively, these findings suggest that most of the unhealthy changes that occurred to adolescents’ 24-h movement behaviours during the early stages of the pandemic were due to the restricted access to schools and sport facilities and that once they were lifted, adolescents quickly returned to their normal movement behaviour levels.

In contrast, the prospective analyses demonstrated considerable changes in the 24-h movement behaviour composition among students who started high school in 2019 and experienced most of it during the pandemic. These changes were characterized by a 17.7% decrease in MVPA, and a 5.1% increase in SB from 2019, when students were in their first high-school year, to 2022, when most students were in their last high-school year. These results are in line with prospective evidence prior to COVID-19 showing that, throughout adolescence, objectively-measured MVPA [28] and SPT [29] tend to decrease while the time spent on SB increases [30]. Taken together, the findings from prospective and repeated cross-sectional analyses suggest that most of the prospective changes in the 24-h movement behaviours occurred as a consequence of aging and the accompanying psychosocial transitions typical of adolescence – such as entering the workforce, increased academic demands, and shifts in leisure-time preferences – and may not have been attributable to COVID-19 restrictions. However, the evidenced unhealthier changes in MVPA [7], SB [9], and SPT [9] from prior-COVID-19 to the earlier stages of the pandemic could not be assessed in this study and, therefore, the investigation comparing the periods prior-, during- and post-COVID-19 should be examined in future studies.

The moderation analysis performed in this study showed that both cross-sectional and longitudinal differences in the 24-h movement behaviour compositions from 2019 to 2022 did not vary according to sociodemographic characteristics. The absence of pronounced moderation effects suggests that external factors, such as the return to in-person schooling, may have played a predominant role in shaping the activity patterns, diminishing the influence of individual sociodemographic characteristics. Sex, age, and SES were previously identified as potential moderators of differences in at least components of the 24-h movement behaviours for comparisons accounting for both early [9] and later [11, 13] phases of the pandemic. However, previous findings were inconsistent. For instance, Hurter and colleagues found an increase in MVPA that was associated with male sex (vs females), higher SES, and lower age among children and adolescents from the UK [11]. Helgadóttir and colleagues examined a sample of Swedish adolescents in 2019 and again in mid-2021 and found that the changes in SPT on weekdays and in LIPA and MVPA during school hours were higher among males than females, but did not differ according parental education [13]. Therefore, moderators of changes in the 24-h movement behaviours accounting for the end of imposing restrictions may be specific to countries and territories. In addition, the limited set of sociodemographic variables included in the moderation analysis restricts the generalizability of the findings. Important contextual and structural factors, such as area of residence (urban vs. rural), neighbourhood deprivation, walkability, and access to green and/or blue spaces, were not captured and may have differentially influenced movement behaviours throughout the pandemic. Therefore, the absence of moderation effects applies only to the investigated individual-level sociodemographic variables, and contextual-level factors should be further explored.

The possibility of the impact of social restrictions on the 24-h movement behaviours lasting for long after the pandemic outbreak among adolescents raises concerns. The absence of school, club, and gym access during the pandemic may have hindered the development of motor skills related to sports, potentially creating barriers for future engagement in physical activity. The increased time spent on screens and the potential expansion of online teaching might have had enduring effects on accelerometer-measured SB. However, the long-term impact of the pandemic was not confirmed in our study. Therefore, the challenges related to the promotion of healthy patterns of 24-h movement behaviours faced before the pandemic seem to largely be the same after the pandemic.

This study has several strengths. First, the repeated cross-sectional design with a nested cohort allowed us to compare cross-sectional and prospective inferences within the same population. The study was conducted in a middle-income country sample, Brazil, and COVD-19 indicated changes in movement behaviours in such countries are scarce. The data collection was conducted a semester prior to COVID-19 in 2019 and in the first semester post the complete reopening of the participant schools, which is an adequate timing to examine pre- and pos-COVID-19 behavioural changes among high-school-aged students. The outcomes (i.e., 24-h movement behaviours) were objectively assessed using accelerometers, which provide more accurate estimates compared to self-reported instruments and allow to account for the compositional structure of the data within the time-use epidemiology framework. The accelerometer-wearing protocol was tested and adapted for the study, which allowed good compliance with the accelerometer criteria (23.8 h/day of average wear time among the analyzed sample). A compositional multilevel modelling approach was applied to consider the co-dependency of the 24-h movement behaviours and the nested nature of data (i.e., participants nested within schools). All the code designed for the statistical analysis was provided, which favours transparency and allows further examination by the scientific community.

This study has limitations that need to be acknowledged. Given the specific contextual factors exclusive to this study’s setting and the large differences in the Brazilian national education systems, the findings of the present study may not extend to adolescents in other regions or school types, particularly those in traditional public high schools or private schools. Data representing the earlier stages of the pandemic when strict restrictive measures were imposed could not be assessed. Although the movement behaviours were assessed based on raw accelerometer data and were processed using open-source algorithms, no behavioural classification cut-off points designed specifically for the age range of the target population (i.e., most aged 15–18 years) are currently available. Thus, a sensitivity analysis was performed to examine whether the findings based on cut-off points designed for children and recommended for adolescents differ from those based on cut-off points designed for adults.

## Conclusions

Both within- and between-participant differences in the 24-h movement behaviour compositions were observed when comparing the periods prior to COVID-19 in 2019 with after the reopening of schools for in-person classes in 2022. Prospective analysis showed a considerable decrease in MVPA and an increase in SB that were expected with aging. No clear pattern of differences was observed for LIPA and SPT. Although in the same direction, the magnitude of differences comparing cross-sectional samples were small. The findings suggest that the 24-h movement behaviours changes were mostly affected by age-related transitions and not by COVID-19 restrictions. Although the negative impact of the early stages of the pandemic on the 24-h movement behaviours, previously identified by several studies, was not observed with the reopening of schools, the pursue of an optimal balance of time use remain as a public health priority.

## Supplementary Information

Additional file 1 includes the design flowcharts (Figures S1 and S2); Comparison between participants who provided valid accelerometer data and those excluded for not complying with the accelerometer protocol in 2019 (Table S1) and in 2022 (Table S2); Comparison between participants who provided valid accelerometer data and those excluded for not complying with the accelerometer protocol in the longitudinal sample (Table S3).

Additional file 2 is an HTML file including the R code and outputs.

Additional file 3 contains the additional findings related with the sensitivity analysis.

## Data Availability

The datasets generated and/or analysed during the current study are not publicly available due to ethical constraints established by the Human Research Ethics Committee of the Federal University of Santa Catarina and the informed consent process, which explicitly stated that individual information would not be publicly shared. However, they are available from the corresponding author upon reasonable request. The R code with detailed description of analyses and outputs, and the report with sensitivity analyses are publicly available in Additional File 2. Additional information on the ELEVA study, including instruments and related publications, are available at https://eleva.ufsc.br/en/.

## Abbreviations

LIPA: Light-intensity physical activity;
MVPA: Moderate-to-vigorous physical activity;
SB: Sedentary behaviour;
SPT: Sleep time;
PVC: Polyvinyl chloride;
ENMO: Euclidean norm minus one;
SES: Socioeconomic status
ILR: Isometric log-ratio
