# Supplementary material for: Assessing the damage: analyzing the impact of the COVID-19 pandemic on accelerometer-assessed 24-hour movement behaviours in Brazilian adolescents
Source: BMC Public Health. 2025 May 31;25:2022. doi: 10.1186/s12889-025-23155-8 (PMC12125940; doi:10.1186/s12889-025-23155-8)
Supplement: Supplementary file 1 — Additional file 1 includes the design flowcharts (Figures S1 and S2); Comparison between participants who provided valid accelerometer data and those excluded for not complying with the accelerometer protocol in 2019 (Table S1) and in 2022 (Table S2); Comparison between participants who provided valid accelerometer data and those excluded for not complying with the accelerometer protocol in the longitudinal sample (Table S3). [file 12889_2025_23155_MOESM1_ESM.pdf]

Additional File 1

Marcus Lopes (mveber@cheo.on.ca)

Additional outputs related to the article ‘Assessing the damage: analyzing the impact of the COVID-19 pandemic on accelerometer-assessed 24-hour movement behaviours in Brazilian adolescents’ authored by Marcus V V Lopes, Ian Janssen, Bruno G G da Costa, Bruno N de Oliveira, Gabrielli T de Mello, Jean-Philippe Chaput, Kelly S Silva

Figure S1. Repeated cross-sectional design of the ELEVA study.

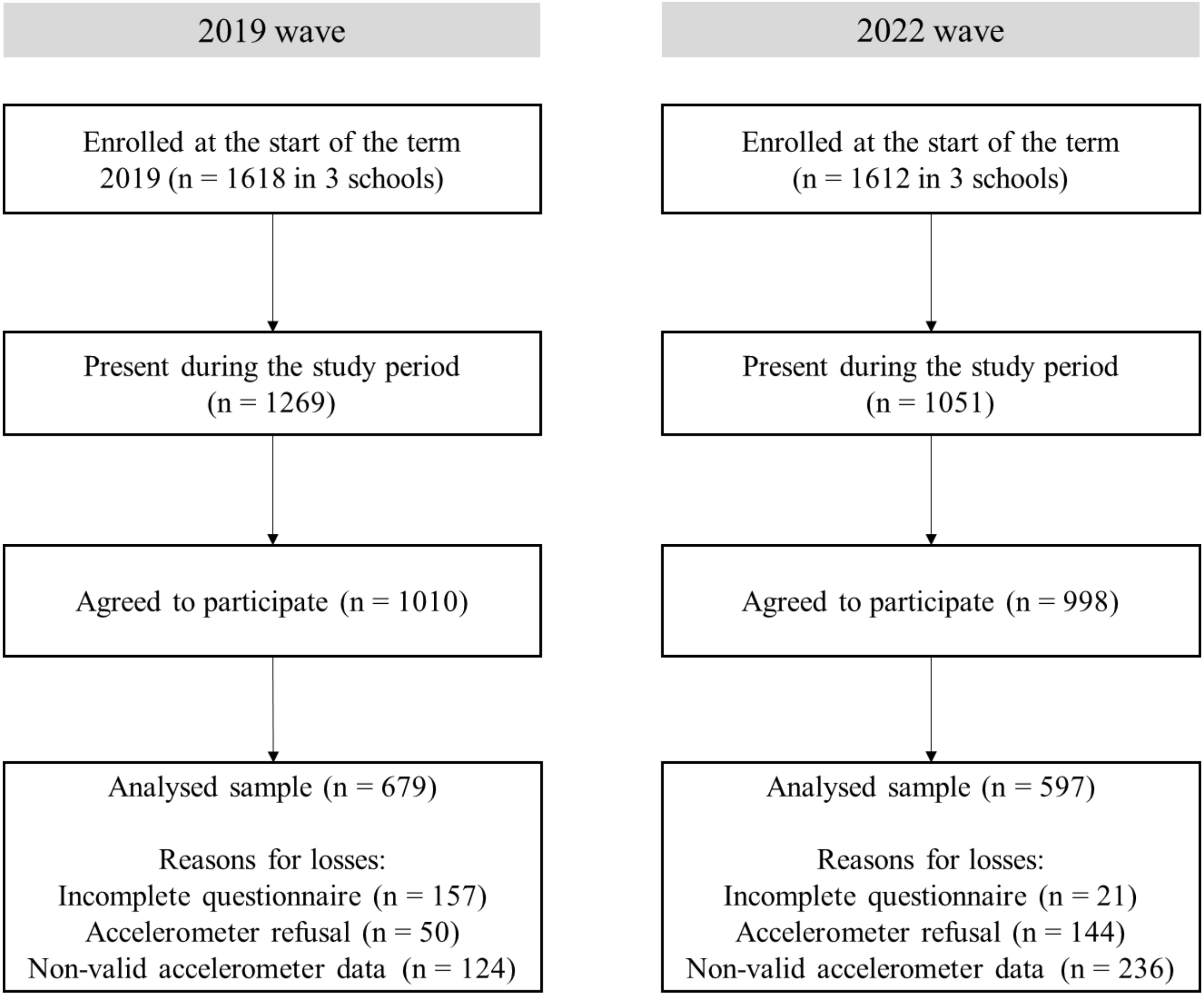

Figure S2. Longitudinal sample within the repeated cross-sectional design of the ELEVA study.

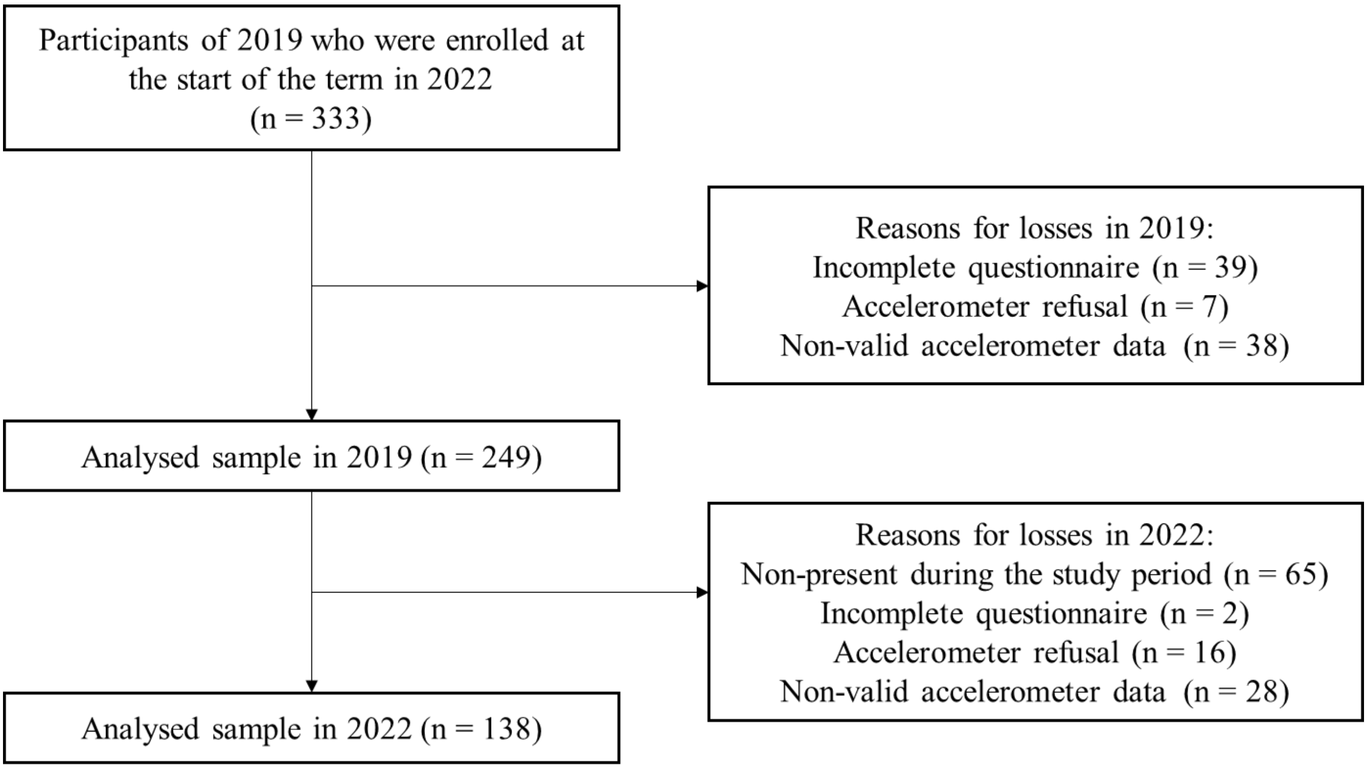

Table S1. Comparison between participants who provided valid accelerometer data and those excluded for not complying with the accelerometer protocol in 2019.

Variable	Excluded, N = 124	Included, N = 679	p-value
**Sex, n (%)**			>0.9
Male	61 (49%)	335 (49%)	
Female	63 (51%)	344 (51%)	
**Age (years), mean (SD)**	16.5 (1.0)	16.3 (1.1)	0.094
**Highest education among parents, n (%)**			0.2
<8 years	3 (2.4%)	40 (5.9%)	
9-11 years	37 (30%)	227 (33%)	
>11 years	83 (67%)	398 (59%)	
Don't know	1 (0.8%)	14 (2.1%)	
**SES score (0 - 100), mean (SD)**	40.5 (10.3)	39.1 (9.8)	
**Family structure, n (%)**			0.6
Live with both parents	74 (60%)	431 (63%)	
Single parent	44 (35%)	212 (31%)	
Doest not live with parents	6 (4.8%)	36 (5.3%)	

Note: [1]Pearson's Chi-squared test; Wilcoxon rank sum test; Fisher's exact test

Table S2. Comparison between participants who provided valid accelerometer data and those excluded for not complying with the accelerometer protocol in 2022.

Variable	Excluded, N = 236	Included, N = 597	p-value
**Sex, n (%)**			0.015
Male	126 (53%)	263 (44%)	
Female	110 (47%)	334 (56%)	
**Age (years), mean (SD)**	16.4 (1.2)	16.5 (1.2)	0.2
**Highest education among parents, n (%)**			0.7
<8 years	11 (4.7%)	33 (5.5%)	
9-11 years	62 (26%)	178 (30%)	
>11 years	153 (65%)	361 (60%)	
Do not know	10 (4.2%)	25 (4.2%)	
**SES score (0 - 100), mean (SD)**	41.2 (10.5)	38.7 (10.2)	
**Family structure, n (%)**			0.7
Live with both parents	141 (60%)	349 (58%)	
Single parent	86 (36%)	218 (37%)	
Do not live with parents	9 (3.8%)	30 (5.0%)	

Note: [1]Pearson's Chi-squared test; Wilcoxon rank sum test; Fisher's exact test

Table S3. Comparison between participants who provided valid accelerometer data and those excluded for not complying with the accelerometer protocol in the longitudinal sample.

Variable	Excluded, N = 38	Included, N = 249	p-value
**Sex, n (%)**			0.8
Male	17 (45%)	117 (47%)	
Female	21 (55%)	132 (53%)	
**Age (years), mean (SD)**	15.6 (0.8)	15.6 (0.8)	0.9
**Highest education among parents, n (%)**			>0.9
<8 years	1 (2.6%)	12 (4.8%)	
9-11 years	13 (34%)	93 (37%)	
>11 years	23 (61%)	137 (55%)	
Do not know	1 (2.6%)	7 (2.8%)	
**SES score (0 - 100), mean (SD)**	38.3 (10.2)	38.5 (9.4)	
**Family structure, n (%)**			0.9
Live with both parents	23 (61%)	159 (64%)	
Single parent	14 (37%)	80 (32%)	
Do not live with parents	1 (2.6%)	10 (4.0%)	

Note: [1]Pearson's Chi-squared test; Wilcoxon rank sum test; Fisher's exact test
