# Supplementary material for: Assessing the damage: analyzing the impact of the COVID-19 pandemic on accelerometer-assessed 24-hour movement behaviours in Brazilian adolescents
Source: BMC Public Health. 2025 May 31;25:2022. doi: 10.1186/s12889-025-23155-8 (PMC12125940; doi:10.1186/s12889-025-23155-8)
Supplement: Supplementary file 2 — Additional file 2 is an HTML file including the R code and outputs. [file 12889_2025_23155_MOESM2_ESM.html]

Code


## Table of contents

- 1 Cross-sectional data
  - 1.1 Visualization of the compositional data
  - 1.2 Preparing data for modeling
  - 1.3 Overall composition difference between time points
  - 1.4 Pivot coordinates differences between time points
  - 1.5 Summary (Table 2)
  - 1.6 Moderation analysis
  - 1.7 Summary (Table 5.1)
- 2 Prospective data
  - 2.1 Visualization of the compositional data
  - 2.2 Preparing data for modeling
  - 2.3 Overall composition difference between time points
  - 2.4 Pivot coordinates differences between time points
  - 2.5 Summary (Table 4)
  - 2.6 Moderation analysis
  - 2.7 Summary (Table 5.2)

# Code

Author

Marcus Lopes (mveber@cheo.on.ca)

Abstract

Code related to the article ‘Assessing the damage: analyzing the impact of the COVID-19 pandemic on accelerometer-assessed 24-hour movement behaviours in Brazilian adolescents’ authored by Marcus V V Lopes, Ian Janssen, Bruno G G da Costa, Bruno N de Oliveira, Gabrielli T de Mello, Jean-Philippe Chaput, Kelly S Silva

```
pacman::p_load(
  tidyverse,
  sjlabelled,
  janitor,
  lme4,
  lmerTest,
  gtsummary,
  compositions,
  robCompositions,
  rgl,
  performance,
  parameters,
  emmeans,
  kableExtra
  )
```

# 1 Cross-sectional data

## 1.1 Visualization of the compositional data

The variables MVPA, LIPA, SB and SPT are declared as a single compositional vector using the `compositional::acomp()` function.

```
labels <- c("MVPA","LIPA","SB","SPT")

coda <- Data_cross %>%
  mutate(
    activity = cbind(MVPA, LIPA, SB, SPT),
    composition = acomp(activity))
```

Compositional outliers based on Robust Mahalanobis Distance are then inspected.

```
outlierplot.acomp(coda$composition, type = 'scatter', robust = T)
```

```
outlierplot.acomp(coda$composition, type = 'biplot', robust = T)
```

```
outliers <- outlierplot.acomp(coda$composition, type = 'distdist')
```

```
outliers <-  data.frame(out_class = outliers[["classes"]],
                        out_mahal = outliers[["NormalMahalanobisDist"]],
                        out_mahal_rob = outliers[["RobustMahalanobisDist"]],
                        out_mahal_crit = outliers[["crit"]])


coda <- bind_cols(coda, outliers) 
coda %>% filter(out_class!='ok') %>% 
  select(MVPA, LIPA, SB, SPT, out_mahal_rob, out_mahal) %>% 
  kbl() %>% kable_styling(bootstrap_options = c("striped", "condensed"), full_width = F)
```

| MVPA | LIPA | SB | SPT | out\_mahal\_rob | out\_mahal |
| --- | --- | --- | --- | --- | --- |
| 19.176 | 559.688 | 379.327 | 481.810 | 5.368026 | 5.007390 |
| 18.396 | 471.762 | 708.699 | 241.143 | 5.449047 | 5.161224 |
| 92.381 | 258.173 | 841.652 | 247.795 | 5.138328 | 4.873112 |
| 2.634 | 163.929 | 657.985 | 615.452 | 5.105974 | 4.782447 |
| 53.758 | 109.563 | 856.238 | 420.440 | 5.362978 | 4.962721 |

Compositional means expressed in proportion relative to the 24 hours

```
coda %>% group_by(survey) %>% 
  reframe(
    avg = mean.acomp(composition),
    beh = labels) %>% 
  ungroup() %>% 
  mutate(avg=round(avg*100,2)) %>% 
  pivot_wider(
    names_from = c(beh),
    values_from = c(avg)
  ) %>% 
  kbl() %>% kable_styling(bootstrap_options = c("striped", "condensed"), full_width = F)
```

| survey | MVPA | LIPA | SB | SPT |
| --- | --- | --- | --- | --- |
| 2019 | 2.13 | 19.24 | 47.19 | 31.43 |
| 2022 | 1.88 | 19.15 | 47.49 | 31.49 |

```
# Variation matrix

var1 <- coda %>% 
  filter(survey=='2019') %>% 
  select(composition) %>% 
  reframe(variation = round(
    compositions::variation(composition), 3)) %>% 
  as.matrix(.) %>% data.frame() %>% 
  mutate(Variables = labels) %>% 
  relocate(Variables)

names(var1) <- c('Variables',labels)
var1 %>% kbl(caption = 'Variation matrix for 2019 data') %>% kable_styling(bootstrap_options = c("striped", "condensed"))
```

Variation matrix for 2019 data

| Variables | MVPA | LIPA | SB | SPT |
| --- | --- | --- | --- | --- |
| MVPA | 0.000 | 0.242 | 0.358 | 0.326 |
| LIPA | 0.242 | 0.000 | 0.097 | 0.074 |
| SB | 0.358 | 0.097 | 0.000 | 0.042 |
| SPT | 0.326 | 0.074 | 0.042 | 0.000 |

```
var2 <- coda %>% 
  filter(survey=='2022') %>% 
  select(composition) %>% 
  reframe(variation = round(
    compositions::variation(composition), 3)) %>% 
  as.matrix(.) %>% data.frame() %>% 
  mutate(Variables = labels) %>% 
  relocate(Variables)

names(var2) <- c('Variables',labels)
var2 %>% kbl(caption = 'Variation matrix for 2022 data') %>% kable_styling(bootstrap_options = c("striped", "condensed"))
```

Variation matrix for 2022 data

| Variables | MVPA | LIPA | SB | SPT |
| --- | --- | --- | --- | --- |
| MVPA | 0.000 | 0.281 | 0.443 | 0.393 |
| LIPA | 0.281 | 0.000 | 0.109 | 0.089 |
| SB | 0.443 | 0.109 | 0.000 | 0.046 |
| SPT | 0.393 | 0.089 | 0.046 | 0.000 |

3D Ternary diagram

```
compositions::plot3D(coda$composition[,1:4], cex=5 ,
                     color=coda$survey, alpha=0.6, axis.col = 'black') 
rglwidget()
```

## 1.2 Preparing data for modeling

First, the pivot coordinates accounting for distinct numerators (i.e., ilr\_mvpa, ilr\_lipa, ilr\_sb, ilr\_spt) are created and stored as data frames. Another coordinate using binary partition is created (i.e., ilr\_simplex). All data frames are merged to the main dataset which will be reshaped to long format.

```
# defining SBP

# creating pivot coordinates
ilr_mvpa <-  coda %>% 
  select(all_of(labels)) %>% 
  data.matrix() %>% 
  pivotCoord(1) %>% 
  data.frame()
names(ilr_mvpa) <- c('ilr_mvpa.1', 'ilr_mvpa.2', 'ilr_mvpa.3')

ilr_lipa <-  coda %>% 
  select(all_of(labels)) %>% 
  data.matrix() %>% 
  pivotCoord(2) %>% 
  data.frame()
names(ilr_lipa) <- c('ilr_lipa.1', 'ilr_lipa.2', 'ilr_lipa.3')

ilr_sb <-  coda %>% 
  select(all_of(labels)) %>% 
  data.matrix() %>% 
  pivotCoord(3) %>% 
  data.frame()
names(ilr_sb) <- c('ilr_sb.1', 'ilr_sb.2', 'ilr_sb.3')

ilr_spt <-  coda %>% 
  select(all_of(labels)) %>% 
  data.matrix() %>% 
  pivotCoord(4) %>% 
  data.frame()
names(ilr_spt) <- c('ilr_spt.1', 'ilr_spt.2', 'ilr_spt.3')

ilr_simplex <-  coda %>% 
  select(all_of(labels)) %>% 
  ilr() %>% data.frame()

coda <- bind_cols(coda, ilr_mvpa, ilr_lipa, ilr_sb, ilr_spt, ilr_simplex) %>% 
  mutate(
    ilr.1 = V1, 
    ilr.2 = V2, 
    ilr.3 = V3
  )

coda_stacked <- coda %>% 
  pivot_longer(cols = starts_with(
    c("ilr.", "ilr_mvpa.", "ilr_lipa.", "ilr_sb.", "ilr_spt.")), 
    names_to = c(".value", "ilr_factor"),
    names_sep = "\\.") %>% 
  mutate(ilr_factor = as_factor(ilr_factor))
```

## 1.3 Overall composition difference between time points

The `lmer` function was used to fit the multilevel model applied to the stacked data. This approach was previously used for modeling compositional data (see (Lim et al. 2023)) and instructions are provided elsewhere (see (Rosen 2023)). The `emmeans` function is used to get adjusted predictions. The `parameters` function is used to extract specific parameters of interest. The stored parameters are used to create the summary table presented in the paper (Section 1.5).

```
model <- lmer(ilr ~  -1 +
                ilr_factor +
                ilr_factor:survey +
                ilr_factor:age +
                ilr_factor:sex +
                ilr_factor:family +
                ilr_factor:ses +
                (1 | campus/id), 
              coda_stacked,
              control = lmerControl(
                optimizer ='optimx', optCtrl=list(method='L-BFGS-B'),
                check.conv.singular = 
                  .makeCC(action = "ignore", 
                          tol = formals(isSingular)$tol)))

check_model(model,  check = c("pp_check", "linearity", "homogeneity", "outliers"))
```

```
check_model(model,  check = c("qq", "normality", "reqq"))
```

```
parameters(model)
```

```
# Fixed Effects

Parameter                                          | Coefficient |       SE
---------------------------------------------------------------------------
ilr factor [1]                                     |        1.35 |     0.14
ilr factor [2]                                     |        1.42 |     0.14
ilr factor [3]                                     |        0.90 |     0.14
ilr factor [1] × survey2022                        |        0.08 |     0.02
ilr factor [2] × survey2022                        |        0.06 |     0.02
ilr factor [3] × survey2022                        |        0.03 |     0.02
ilr factor [1] × age                               |    9.99e-03 | 7.82e-03
ilr factor [2] × age                               |        0.01 | 7.82e-03
ilr factor [3] × age                               |   -8.00e-03 | 7.82e-03
ilr factor [1] × sexFemale                         |        0.14 |     0.02
ilr factor [2] × sexFemale                         |       -0.02 |     0.02
ilr factor [3] × sexFemale                         |        0.06 |     0.02
ilr factor [1] × familySingle parent               |       -0.06 |     0.02
ilr factor [2] × familySingle parent               |   -6.17e-03 |     0.02
ilr factor [3] × familySingle parent               |   -3.92e-03 |     0.02
ilr factor [1] × familyDoest not live with parents |       -0.05 |     0.04
ilr factor [2] × familyDoest not live with parents |       -0.05 |     0.04
ilr factor [3] × familyDoest not live with parents |    9.81e-03 |     0.04
ilr factor [1] × ses                               |    1.64e-04 | 9.32e-04
ilr factor [2] × ses                               |    6.84e-04 | 9.32e-04
ilr factor [3] × ses                               |    2.68e-04 | 9.32e-04

Parameter                                          |         95% CI | t(3804) |      p
--------------------------------------------------------------------------------------
ilr factor [1]                                     | [ 1.08,  1.62] |    9.85 | < .001
ilr factor [2]                                     | [ 1.15,  1.69] |   10.36 | < .001
ilr factor [3]                                     | [ 0.64,  1.17] |    6.60 | < .001
ilr factor [1] × survey2022                        | [ 0.04,  0.11] |    4.39 | < .001
ilr factor [2] × survey2022                        | [ 0.02,  0.09] |    3.13 | 0.002 
ilr factor [3] × survey2022                        | [ 0.00,  0.07] |    1.93 | 0.054 
ilr factor [1] × age                               | [-0.01,  0.03] |    1.28 | 0.201 
ilr factor [2] × age                               | [ 0.00,  0.03] |    1.65 | 0.099 
ilr factor [3] × age                               | [-0.02,  0.01] |   -1.02 | 0.307 
ilr factor [1] × sexFemale                         | [ 0.10,  0.17] |    7.51 | < .001
ilr factor [2] × sexFemale                         | [-0.05,  0.02] |   -0.96 | 0.339 
ilr factor [3] × sexFemale                         | [ 0.03,  0.10] |    3.34 | < .001
ilr factor [1] × familySingle parent               | [-0.09, -0.02] |   -2.79 | 0.005 
ilr factor [2] × familySingle parent               | [-0.04,  0.03] |   -0.31 | 0.755 
ilr factor [3] × familySingle parent               | [-0.04,  0.03] |   -0.20 | 0.843 
ilr factor [1] × familyDoest not live with parents | [-0.13,  0.03] |   -1.25 | 0.213 
ilr factor [2] × familyDoest not live with parents | [-0.14,  0.03] |   -1.32 | 0.186 
ilr factor [3] × familyDoest not live with parents | [-0.07,  0.09] |    0.24 | 0.812 
ilr factor [1] × ses                               | [ 0.00,  0.00] |    0.18 | 0.861 
ilr factor [2] × ses                               | [ 0.00,  0.00] |    0.73 | 0.463 
ilr factor [3] × ses                               | [ 0.00,  0.00] |    0.29 | 0.773 

# Random Effects

Parameter                 | Coefficient
---------------------------------------
SD (Intercept: id:campus) |        0.26
SD (Intercept: campus)    |        0.04
SD (Residual)             |        0.19
```

```
parameters(car::Anova(model, type = "III"))
```

```
Parameter         |   Chi2 | df |      p
----------------------------------------
ilr_factor        | 127.74 |  3 | < .001
ilr_factor:survey |  21.67 |  3 | < .001
ilr_factor:age    |  12.62 |  3 | 0.006 
ilr_factor:sex    | 119.65 |  3 | < .001
ilr_factor:family |  17.95 |  6 | 0.006 
ilr_factor:ses    |   0.71 |  3 | 0.870 

Anova Table (Type 3 tests)
```

```
comp <- parameters(car::Anova(model, type = "III")) %>% 
  filter(Parameter=='ilr_factor:survey') %>% 
  select(p) %>% 
  mutate(
    parameter = "Composition",
    p = sprintf(p, fmt = "%.3f")) %>% 
  clean_names() %>% 
  as_tibble()

pred <- tidy(emmeans::emmeans(model, ~ ilr_factor*survey, weights = "proportional")) %>%
  select(c("estimate", 'std.error', 'survey', 'ilr_factor')) %>% 
  pivot_wider(names_from = 'ilr_factor', values_from =c("estimate", "std.error")) %>% 
  # Change to include CI
  select(survey, c(starts_with('estimate'))) %>% 
  mutate(geo = clo(ilrInv(cbind(
    `estimate_1`, 
    `estimate_2`, 
    `estimate_3`)), total=1440),
    geo = round(geo, 1))

pred <- data.frame(pred$geo,
                   survey = pred$survey) %>% 
  relocate(survey)

colnames(pred)[2:5] <- labels 

pred <- transpose(pred, make.names = 1)
pred <- rbind(NA, pred)
```

## 1.4 Pivot coordinates differences between time points

The second set of models were fitted using the pivot coordinates as dependent variables. The `ilr_factor1:survey` coefficient is interpreted as the differences between surveys for the relative contribution of the numerator to the remaining behaviors.

```
outcomes <- c('ilr_mvpa', 'ilr_lipa', 'ilr_sb', 'ilr_spt')

# Running the models and saving parameters
for (outcome in outcomes) {
  
  model <- lmer(get(outcome) ~ -1 +
                  ilr_factor +
                  ilr_factor:survey +
                  ilr_factor:age +
                  ilr_factor:sex +
                  ilr_factor:family +
                  ilr_factor:ses +
                  (1 | campus/id), 
                coda_stacked,
                control = lmerControl(
                  optimizer ='optimx', optCtrl=list(method='L-BFGS-B'),
                  check.conv.singular = 
                    .makeCC(action = "ignore", 
                            tol = formals(isSingular)$tol)))
  print(' ')
  print(outcome)
  parameters(model) %>% print()
  
  coef <- parameters(model) %>% 
    filter(Parameter=='ilr_factor1:survey2022') %>% 
    mutate(
      Coefficient = sprintf(Coefficient, fmt = "%.3f"),
      p = sprintf(p, fmt = "%.3f")) %>% 
    clean_names() %>% 
    select(c('parameter', 'coefficient', 'p')) %>% 
    as_tibble() 
  
  assign(paste0('m1_', outcome), model)
  assign(paste0('c1_', outcome), coef)
  
  remove(list = c('model'))
}
```

```
[1] " "
[1] "ilr_mvpa"
# Fixed Effects

Parameter                                          | Coefficient |       SE
---------------------------------------------------------------------------
ilr factor [1]                                     |       -2.07 |     0.14
ilr factor [2]                                     |       -0.59 |     0.14
ilr factor [3]                                     |        0.08 |     0.14
ilr factor [1] × survey2022                        |       -0.10 |     0.02
ilr factor [2] × survey2022                        |   -7.51e-03 |     0.02
ilr factor [3] × survey2022                        |    5.79e-03 |     0.02
ilr factor [1] × age                               |       -0.01 | 7.81e-03
ilr factor [2] × age                               |    7.49e-04 | 7.81e-03
ilr factor [3] × age                               |        0.01 | 7.81e-03
ilr factor [1] × sexFemale                         |       -0.12 |     0.02
ilr factor [2] × sexFemale                         |        0.06 |     0.02
ilr factor [3] × sexFemale                         |       -0.06 |     0.02
ilr factor [1] × familySingle parent               |        0.05 |     0.02
ilr factor [2] × familySingle parent               |       -0.03 |     0.02
ilr factor [3] × familySingle parent               |   -3.43e-04 |     0.02
ilr factor [1] × familyDoest not live with parents |        0.06 |     0.04
ilr factor [2] × familyDoest not live with parents |    1.91e-03 |     0.04
ilr factor [3] × familyDoest not live with parents |       -0.04 |     0.04
ilr factor [1] × ses                               |   -3.31e-04 | 9.30e-04
ilr factor [2] × ses                               |   -6.20e-04 | 9.30e-04
ilr factor [3] × ses                               |   -6.19e-05 | 9.30e-04

Parameter                                          |         95% CI | t(3804) |      p
--------------------------------------------------------------------------------------
ilr factor [1]                                     | [-2.34, -1.80] |  -15.14 | < .001
ilr factor [2]                                     | [-0.86, -0.32] |   -4.32 | < .001
ilr factor [3]                                     | [-0.18,  0.35] |    0.62 | 0.537 
ilr factor [1] × survey2022                        | [-0.14, -0.07] |   -5.81 | < .001
ilr factor [2] × survey2022                        | [-0.04,  0.03] |   -0.42 | 0.675 
ilr factor [3] × survey2022                        | [-0.03,  0.04] |    0.32 | 0.747 
ilr factor [1] × age                               | [-0.03,  0.00] |   -1.49 | 0.137 
ilr factor [2] × age                               | [-0.01,  0.02] |    0.10 | 0.924 
ilr factor [3] × age                               | [ 0.00,  0.03] |    1.76 | 0.078 
ilr factor [1] × sexFemale                         | [-0.16, -0.09] |   -6.93 | < .001
ilr factor [2] × sexFemale                         | [ 0.03,  0.10] |    3.42 | < .001
ilr factor [3] × sexFemale                         | [-0.09, -0.02] |   -3.26 | 0.001 
ilr factor [1] × familySingle parent               | [ 0.01,  0.09] |    2.49 | 0.013 
ilr factor [2] × familySingle parent               | [-0.06,  0.01] |   -1.31 | 0.190 
ilr factor [3] × familySingle parent               | [-0.04,  0.04] |   -0.02 | 0.986 
ilr factor [1] × familyDoest not live with parents | [-0.02,  0.14] |    1.54 | 0.124 
ilr factor [2] × familyDoest not live with parents | [-0.08,  0.08] |    0.05 | 0.963 
ilr factor [3] × familyDoest not live with parents | [-0.12,  0.04] |   -0.96 | 0.339 
ilr factor [1] × ses                               | [ 0.00,  0.00] |   -0.36 | 0.722 
ilr factor [2] × ses                               | [ 0.00,  0.00] |   -0.67 | 0.505 
ilr factor [3] × ses                               | [ 0.00,  0.00] |   -0.07 | 0.947 

# Random Effects

Parameter                 | Coefficient
---------------------------------------
SD (Intercept: id:campus) |        0.10
SD (Intercept: campus)    |        0.04
SD (Residual)             |        0.30
```

```
[1] " "
[1] "ilr_lipa"
# Fixed Effects

Parameter                                          | Coefficient |       SE
---------------------------------------------------------------------------
ilr factor [1]                                     |        0.14 |     0.14
ilr factor [2]                                     |       -2.15 |     0.14
ilr factor [3]                                     |        0.09 |     0.14
ilr factor [1] × survey2022                        |        0.03 |     0.02
ilr factor [2] × survey2022                        |       -0.10 |     0.02
ilr factor [3] × survey2022                        |    5.25e-03 |     0.02
ilr factor [1] × age                               |    4.35e-03 | 7.82e-03
ilr factor [2] × age                               |       -0.01 | 7.82e-03
ilr factor [3] × age                               |        0.01 | 7.82e-03
ilr factor [1] × sexFemale                         |        0.10 |     0.02
ilr factor [2] × sexFemale                         |       -0.10 |     0.02
ilr factor [3] × sexFemale                         |       -0.06 |     0.02
ilr factor [1] × familySingle parent               |       -0.04 |     0.02
ilr factor [2] × familySingle parent               |        0.04 |     0.02
ilr factor [3] × familySingle parent               |   -4.10e-04 |     0.02
ilr factor [1] × familyDoest not live with parents |       -0.02 |     0.04
ilr factor [2] × familyDoest not live with parents |        0.06 |     0.04
ilr factor [3] × familyDoest not live with parents |       -0.04 |     0.04
ilr factor [1] × ses                               |   -5.50e-04 | 9.30e-04
ilr factor [2] × ses                               |   -3.85e-04 | 9.30e-04
ilr factor [3] × ses                               |   -1.47e-05 | 9.30e-04

Parameter                                          |         95% CI | t(3804) |      p
--------------------------------------------------------------------------------------
ilr factor [1]                                     | [-0.13,  0.41] |    1.01 | 0.314 
ilr factor [2]                                     | [-2.41, -1.88] |  -15.73 | < .001
ilr factor [3]                                     | [-0.18,  0.35] |    0.64 | 0.525 
ilr factor [1] × survey2022                        | [-0.01,  0.06] |    1.55 | 0.120 
ilr factor [2] × survey2022                        | [-0.14, -0.07] |   -5.67 | < .001
ilr factor [3] × survey2022                        | [-0.03,  0.04] |    0.29 | 0.770 
ilr factor [1] × age                               | [-0.01,  0.02] |    0.56 | 0.578 
ilr factor [2] × age                               | [-0.03,  0.00] |   -1.38 | 0.168 
ilr factor [3] × age                               | [ 0.00,  0.03] |    1.74 | 0.081 
ilr factor [1] × sexFemale                         | [ 0.06,  0.13] |    5.51 | < .001
ilr factor [2] × sexFemale                         | [-0.13, -0.06] |   -5.46 | < .001
ilr factor [3] × sexFemale                         | [-0.09, -0.02] |   -3.32 | < .001
ilr factor [1] × familySingle parent               | [-0.08,  0.00] |   -2.06 | 0.039 
ilr factor [2] × familySingle parent               | [ 0.00,  0.08] |    1.90 | 0.057 
ilr factor [3] × familySingle parent               | [-0.04,  0.04] |   -0.02 | 0.983 
ilr factor [1] × familyDoest not live with parents | [-0.10,  0.06] |   -0.48 | 0.630 
ilr factor [2] × familyDoest not live with parents | [-0.02,  0.14] |    1.45 | 0.147 
ilr factor [3] × familyDoest not live with parents | [-0.12,  0.04] |   -0.97 | 0.332 
ilr factor [1] × ses                               | [ 0.00,  0.00] |   -0.59 | 0.554 
ilr factor [2] × ses                               | [ 0.00,  0.00] |   -0.41 | 0.679 
ilr factor [3] × ses                               | [ 0.00,  0.00] |   -0.02 | 0.987 

# Random Effects

Parameter                 | Coefficient
---------------------------------------
SD (Intercept: id:campus) |        0.00
SD (Intercept: campus)    |        0.03
SD (Residual)             |        0.32
```

```
[1] " "
[1] "ilr_sb"
# Fixed Effects

Parameter                                          | Coefficient |       SE
---------------------------------------------------------------------------
ilr factor [1]                                     |        1.04 |     0.14
ilr factor [2]                                     |       -1.83 |     0.14
ilr factor [3]                                     |       -0.48 |     0.14
ilr factor [1] × survey2022                        |        0.04 |     0.02
ilr factor [2] × survey2022                        |       -0.10 |     0.02
ilr factor [3] × survey2022                        |   -4.74e-03 |     0.02
ilr factor [1] × age                               |        0.01 | 7.83e-03
ilr factor [2] × age                               |   -6.75e-03 | 7.83e-03
ilr factor [3] × age                               |    7.97e-03 | 7.83e-03
ilr factor [1] × sexFemale                         |       -0.03 |     0.02
ilr factor [2] × sexFemale                         |       -0.14 |     0.02
ilr factor [3] × sexFemale                         |        0.02 |     0.02
ilr factor [1] × familySingle parent               |   -4.33e-03 |     0.02
ilr factor [2] × familySingle parent               |        0.05 |     0.02
ilr factor [3] × familySingle parent               |       -0.02 |     0.02
ilr factor [1] × familyDoest not live with parents |       -0.05 |     0.04
ilr factor [2] × familyDoest not live with parents |        0.05 |     0.04
ilr factor [3] × familyDoest not live with parents |       -0.02 |     0.04
ilr factor [1] × ses                               |    1.48e-04 | 9.32e-04
ilr factor [2] × ses                               |   -9.85e-05 | 9.32e-04
ilr factor [3] × ses                               |   -3.45e-04 | 9.32e-04

Parameter                                          |         95% CI | t(3804) |      p
--------------------------------------------------------------------------------------
ilr factor [1]                                     | [ 0.77,  1.30] |    7.60 | < .001
ilr factor [2]                                     | [-2.10, -1.57] |  -13.48 | < .001
ilr factor [3]                                     | [-0.74, -0.21] |   -3.49 | < .001
ilr factor [1] × survey2022                        | [ 0.01,  0.08] |    2.49 | 0.013 
ilr factor [2] × survey2022                        | [-0.13, -0.06] |   -5.32 | < .001
ilr factor [3] × survey2022                        | [-0.04,  0.03] |   -0.26 | 0.792 
ilr factor [1] × age                               | [ 0.00,  0.03] |    1.90 | 0.058 
ilr factor [2] × age                               | [-0.02,  0.01] |   -0.86 | 0.389 
ilr factor [3] × age                               | [-0.01,  0.02] |    1.02 | 0.309 
ilr factor [1] × sexFemale                         | [-0.07,  0.00] |   -1.86 | 0.063 
ilr factor [2] × sexFemale                         | [-0.18, -0.11] |   -7.99 | < .001
ilr factor [3] × sexFemale                         | [-0.01,  0.06] |    1.36 | 0.175 
ilr factor [1] × familySingle parent               | [-0.04,  0.03] |   -0.22 | 0.827 
ilr factor [2] × familySingle parent               | [ 0.01,  0.09] |    2.55 | 0.011 
ilr factor [3] × familySingle parent               | [-0.06,  0.02] |   -1.14 | 0.256 
ilr factor [1] × familyDoest not live with parents | [-0.13,  0.03] |   -1.29 | 0.198 
ilr factor [2] × familyDoest not live with parents | [-0.03,  0.13] |    1.18 | 0.236 
ilr factor [3] × familyDoest not live with parents | [-0.10,  0.06] |   -0.42 | 0.675 
ilr factor [1] × ses                               | [ 0.00,  0.00] |    0.16 | 0.874 
ilr factor [2] × ses                               | [ 0.00,  0.00] |   -0.11 | 0.916 
ilr factor [3] × ses                               | [ 0.00,  0.00] |   -0.37 | 0.711 

# Random Effects

Parameter                 | Coefficient
---------------------------------------
SD (Intercept: id:campus) |        0.00
SD (Intercept: campus)    |        0.02
SD (Residual)             |        0.32
```

```
[1] " "
[1] "ilr_spt"
# Fixed Effects

Parameter                                          | Coefficient |       SE
---------------------------------------------------------------------------
ilr factor [1]                                     |        0.90 |     0.14
ilr factor [2]                                     |       -1.88 |     0.14
ilr factor [3]                                     |       -0.55 |     0.14
ilr factor [1] × survey2022                        |        0.04 |     0.02
ilr factor [2] × survey2022                        |       -0.10 |     0.02
ilr factor [3] × survey2022                        |       -0.01 |     0.02
ilr factor [1] × age                               |   -8.12e-03 | 7.84e-03
ilr factor [2] × age                               |       -0.01 | 7.84e-03
ilr factor [3] × age                               |   -6.14e-03 | 7.84e-03
ilr factor [1] × sexFemale                         |        0.06 |     0.02
ilr factor [2] × sexFemale                         |       -0.11 |     0.02
ilr factor [3] × sexFemale                         |        0.08 |     0.02
ilr factor [1] × familySingle parent               |   -3.83e-03 |     0.02
ilr factor [2] × familySingle parent               |        0.05 |     0.02
ilr factor [3] × familySingle parent               |       -0.02 |     0.02
ilr factor [1] × familyDoest not live with parents |        0.01 |     0.04
ilr factor [2] × familyDoest not live with parents |        0.07 |     0.04
ilr factor [3] × familyDoest not live with parents |        0.02 |     0.04
ilr factor [1] × ses                               |   -5.83e-05 | 9.32e-04
ilr factor [2] × ses                               |   -3.72e-05 | 9.32e-04
ilr factor [3] × ses                               |   -3.91e-04 | 9.32e-04

Parameter                                          |         95% CI | t(3804) |      p
--------------------------------------------------------------------------------------
ilr factor [1]                                     | [ 0.64,  1.17] |    6.67 | < .001
ilr factor [2]                                     | [-2.15, -1.61] |  -13.86 | < .001
ilr factor [3]                                     | [-0.82, -0.29] |   -4.09 | < .001
ilr factor [1] × survey2022                        | [ 0.00,  0.07] |    2.05 | 0.040 
ilr factor [2] × survey2022                        | [-0.13, -0.06] |   -5.54 | < .001
ilr factor [3] × survey2022                        | [-0.05,  0.03] |   -0.57 | 0.572 
ilr factor [1] × age                               | [-0.02,  0.01] |   -1.04 | 0.300 
ilr factor [2] × age                               | [-0.03,  0.00] |   -1.91 | 0.057 
ilr factor [3] × age                               | [-0.02,  0.01] |   -0.78 | 0.434 
ilr factor [1] × sexFemale                         | [ 0.03,  0.10] |    3.46 | < .001
ilr factor [2] × sexFemale                         | [-0.15, -0.08] |   -6.20 | < .001
ilr factor [3] × sexFemale                         | [ 0.05,  0.12] |    4.58 | < .001
ilr factor [1] × familySingle parent               | [-0.04,  0.04] |   -0.19 | 0.847 
ilr factor [2] × familySingle parent               | [ 0.01,  0.09] |    2.55 | 0.011 
ilr factor [3] × familySingle parent               | [-0.06,  0.02] |   -1.12 | 0.262 
ilr factor [1] × familyDoest not live with parents | [-0.07,  0.09] |    0.25 | 0.800 
ilr factor [2] × familyDoest not live with parents | [-0.01,  0.15] |    1.71 | 0.087 
ilr factor [3] × familyDoest not live with parents | [-0.06,  0.10] |    0.51 | 0.607 
ilr factor [1] × ses                               | [ 0.00,  0.00] |   -0.06 | 0.950 
ilr factor [2] × ses                               | [ 0.00,  0.00] |   -0.04 | 0.968 
ilr factor [3] × ses                               | [ 0.00,  0.00] |   -0.42 | 0.675 

# Random Effects

Parameter                 | Coefficient
---------------------------------------
SD (Intercept: id:campus) |        0.00
SD (Intercept: campus)    |        0.00
SD (Residual)             |        0.32
```

```
# Combining parameters in a single object

coef <- bind_rows(c1_ilr_mvpa, c1_ilr_lipa, c1_ilr_sb, c1_ilr_spt) %>% 
  mutate(parameter = labels) %>% 
  bind_rows(comp) 

coef <- coef[c(5,1,2,3,4), ]
```

## 1.5 Summary (Table 2)

Parameters of interest from the models

```
tbl2 <- bind_cols(pred, coef) %>% 
  mutate(
    p = as.character(p),
    p = case_match(
      p,
      '0.000' ~ '<0.001',
      .default = p),
    change =  sprintf((`2022`/`2019`-1)*100, fmt = "%.1f")) %>% 
  relocate(parameter, `2019`, `2022`, change) 

tbl2 %>% 
  kbl(align = 'c',
      col.names = c('Outcomes', '2019 Pred.avg', '2022 Pred.avg', 'Difference (%)', 'Coef', 'p')) %>% 
  kable_styling(bootstrap_options = c("striped", "hover", "condensed", "responsive"))
```

| Outcomes | 2019 Pred.avg | 2022 Pred.avg | Difference (%) | Coef | p |
| --- | --- | --- | --- | --- | --- |
| Composition | NA | NA | NA | NA | <0.001 |
| MVPA | 29.9 | 26.6 | -11.0 | -0.104 | <0.001 |
| LIPA | 275.8 | 274.0 | -0.7 | 0.028 | 0.120 |
| SB | 679.6 | 684.3 | 0.7 | 0.045 | 0.013 |
| SPT | 454.7 | 455.1 | 0.1 | 0.037 | 0.040 |

## 1.6 Moderation analysis

The third set of models were fitted to test whether the effect of time point on the composition varied according to sociodemographic characteristics.

```
moderators <- c('sex', 'age', 'family', 'ses')

for (moderator in moderators) {
  
  formula <- paste0('ilr ~ -1 +
                  ilr_factor +
                  ilr_factor:survey +
                  ilr_factor:sex +
                  ilr_factor:age +
                  ilr_factor:family +
                  ilr_factor:ses +
                  ilr_factor:survey:', 
                  moderator,
                  ' + (1 | campus/id)')
  
  model <- lmer(formula, 
                coda_stacked,
                control = lmerControl(
                  optimizer ='optimx', optCtrl=list(method='L-BFGS-B'),
                  check.conv.singular = 
                    .makeCC(action = "ignore", 
                            tol = formals(isSingular)$tol)))
  print(' ')
  print(moderator)
  parameters(model) %>% print()
  
  parameters(car::Anova(model, type = "III")) %>% 
    print()
  
  effect <- parameters(car::Anova(model, type = "III")) %>% 
    filter(Parameter == paste0('ilr_factor:survey:', moderator)) %>% 
    select(Parameter, df, Chi2, p) %>% 
    mutate(
      Parameter = paste0('ilr_factor * survey * ', moderator),
      p = sprintf(p, fmt = "%.3f")) %>% 
    clean_names() %>% 
    as_tibble()
  
  assign(paste0('inter_', moderator), effect)
  remove(list = c('effect', 'model'))
}
```

```
[1] " "
[1] "sex"
# Fixed Effects

Parameter                                          | Coefficient |       SE
---------------------------------------------------------------------------
ilr factor [1]                                     |        1.34 |     0.14
ilr factor [2]                                     |        1.41 |     0.14
ilr factor [3]                                     |        0.90 |     0.14
ilr factor [1] × survey2022                        |        0.11 |     0.03
ilr factor [2] × survey2022                        |        0.07 |     0.03
ilr factor [3] × survey2022                        |        0.05 |     0.03
ilr factor [1] × sexFemale                         |        0.16 |     0.02
ilr factor [2] × sexFemale                         |   -1.08e-03 |     0.02
ilr factor [3] × sexFemale                         |        0.07 |     0.02
ilr factor [1] × age                               |    9.50e-03 | 7.83e-03
ilr factor [2] × age                               |        0.01 | 7.83e-03
ilr factor [3] × age                               |   -8.21e-03 | 7.83e-03
ilr factor [1] × familySingle parent               |       -0.05 |     0.02
ilr factor [2] × familySingle parent               |   -5.33e-03 |     0.02
ilr factor [3] × familySingle parent               |   -3.35e-03 |     0.02
ilr factor [1] × familyDoest not live with parents |       -0.05 |     0.04
ilr factor [2] × familyDoest not live with parents |       -0.05 |     0.04
ilr factor [3] × familyDoest not live with parents |        0.01 |     0.04
ilr factor [1] × ses                               |    2.40e-04 | 9.33e-04
ilr factor [2] × ses                               |    7.38e-04 | 9.33e-04
ilr factor [3] × ses                               |    3.08e-04 | 9.33e-04
ilr factor [1] × survey2022 × sexFemale            |       -0.05 |     0.04
ilr factor [2] × survey2022 × sexFemale            |       -0.03 |     0.04
ilr factor [3] × survey2022 × sexFemale            |       -0.02 |     0.04

Parameter                                          |         95% CI | t(3801) |      p
--------------------------------------------------------------------------------------
ilr factor [1]                                     | [ 1.07,  1.61] |    9.79 | < .001
ilr factor [2]                                     | [ 1.14,  1.68] |   10.31 | < .001
ilr factor [3]                                     | [ 0.63,  1.17] |    6.56 | < .001
ilr factor [1] × survey2022                        | [ 0.05,  0.16] |    4.05 | < .001
ilr factor [2] × survey2022                        | [ 0.02,  0.13] |    2.85 | 0.004 
ilr factor [3] × survey2022                        | [ 0.00,  0.10] |    1.79 | 0.073 
ilr factor [1] × sexFemale                         | [ 0.11,  0.21] |    6.50 | < .001
ilr factor [2] × sexFemale                         | [-0.05,  0.05] |   -0.04 | 0.965 
ilr factor [3] × sexFemale                         | [ 0.02,  0.12] |    2.90 | 0.004 
ilr factor [1] × age                               | [-0.01,  0.02] |    1.21 | 0.225 
ilr factor [2] × age                               | [ 0.00,  0.03] |    1.61 | 0.108 
ilr factor [3] × age                               | [-0.02,  0.01] |   -1.05 | 0.294 
ilr factor [1] × familySingle parent               | [-0.09, -0.02] |   -2.73 | 0.006 
ilr factor [2] × familySingle parent               | [-0.04,  0.03] |   -0.27 | 0.787 
ilr factor [3] × familySingle parent               | [-0.04,  0.04] |   -0.17 | 0.865 
ilr factor [1] × familyDoest not live with parents | [-0.13,  0.03] |   -1.20 | 0.229 
ilr factor [2] × familyDoest not live with parents | [-0.13,  0.03] |   -1.29 | 0.196 
ilr factor [3] × familyDoest not live with parents | [-0.07,  0.09] |    0.26 | 0.798 
ilr factor [1] × ses                               | [ 0.00,  0.00] |    0.26 | 0.797 
ilr factor [2] × ses                               | [ 0.00,  0.00] |    0.79 | 0.429 
ilr factor [3] × ses                               | [ 0.00,  0.00] |    0.33 | 0.741 
ilr factor [1] × survey2022 × sexFemale            | [-0.12,  0.02] |   -1.44 | 0.150 
ilr factor [2] × survey2022 × sexFemale            | [-0.11,  0.04] |   -0.97 | 0.332 
ilr factor [3] × survey2022 × sexFemale            | [-0.09,  0.05] |   -0.66 | 0.511 

# Random Effects

Parameter                 | Coefficient
---------------------------------------
SD (Intercept: id:campus) |        0.26
SD (Intercept: campus)    |        0.04
SD (Residual)             |        0.19
```

```
Parameter             |   Chi2 | df |      p
--------------------------------------------
ilr_factor            | 126.29 |  3 | < .001
ilr_factor:survey     |  18.25 |  3 | < .001
ilr_factor:sex        |  75.26 |  3 | < .001
ilr_factor:age        |  12.31 |  3 | 0.006 
ilr_factor:family     |  17.45 |  6 | 0.008 
ilr_factor:ses        |   0.76 |  3 | 0.859 
ilr_factor:survey:sex |   2.26 |  3 | 0.520 

Anova Table (Type 3 tests)
```

```
[1] " "
[1] "age"
# Fixed Effects

Parameter                                          | Coefficient |       SE
---------------------------------------------------------------------------
ilr factor [1]                                     |        1.53 |     0.19
ilr factor [2]                                     |        1.51 |     0.19
ilr factor [3]                                     |        0.92 |     0.19
ilr factor [1] × survey2022                        |       -0.26 |     0.26
ilr factor [2] × survey2022                        |       -0.12 |     0.26
ilr factor [3] × survey2022                        |    9.39e-03 |     0.26
ilr factor [1] × sexFemale                         |        0.14 |     0.02
ilr factor [2] × sexFemale                         |       -0.02 |     0.02
ilr factor [3] × sexFemale                         |        0.06 |     0.02
ilr factor [1] × age                               |   -1.03e-03 |     0.01
ilr factor [2] × age                               |    7.20e-03 |     0.01
ilr factor [3] × age                               |   -8.80e-03 |     0.01
ilr factor [1] × familySingle parent               |       -0.05 |     0.02
ilr factor [2] × familySingle parent               |   -5.55e-03 |     0.02
ilr factor [3] × familySingle parent               |   -3.83e-03 |     0.02
ilr factor [1] × familyDoest not live with parents |       -0.05 |     0.04
ilr factor [2] × familyDoest not live with parents |       -0.06 |     0.04
ilr factor [3] × familyDoest not live with parents |    9.63e-03 |     0.04
ilr factor [1] × ses                               |    1.52e-04 | 9.32e-04
ilr factor [2] × ses                               |    6.75e-04 | 9.32e-04
ilr factor [3] × ses                               |    2.63e-04 | 9.32e-04
ilr factor [1] × survey2022 × age                  |        0.02 |     0.02
ilr factor [2] × survey2022 × age                  |        0.01 |     0.02
ilr factor [3] × survey2022 × age                  |    1.53e-03 |     0.02

Parameter                                          |         95% CI | t(3801) |      p
--------------------------------------------------------------------------------------
ilr factor [1]                                     | [ 1.15,  1.90] |    7.98 | < .001
ilr factor [2]                                     | [ 1.14,  1.89] |    7.89 | < .001
ilr factor [3]                                     | [ 0.54,  1.29] |    4.78 | < .001
ilr factor [1] × survey2022                        | [-0.77,  0.24] |   -1.03 | 0.303 
ilr factor [2] × survey2022                        | [-0.63,  0.38] |   -0.47 | 0.636 
ilr factor [3] × survey2022                        | [-0.50,  0.51] |    0.04 | 0.971 
ilr factor [1] × sexFemale                         | [ 0.10,  0.17] |    7.56 | < .001
ilr factor [2] × sexFemale                         | [-0.05,  0.02] |   -0.92 | 0.356 
ilr factor [3] × sexFemale                         | [ 0.03,  0.10] |    3.35 | < .001
ilr factor [1] × age                               | [-0.02,  0.02] |   -0.09 | 0.928 
ilr factor [2] × age                               | [-0.02,  0.03] |    0.63 | 0.526 
ilr factor [3] × age                               | [-0.03,  0.01] |   -0.78 | 0.438 
ilr factor [1] × familySingle parent               | [-0.09, -0.02] |   -2.73 | 0.006 
ilr factor [2] × familySingle parent               | [-0.04,  0.03] |   -0.28 | 0.779 
ilr factor [3] × familySingle parent               | [-0.04,  0.03] |   -0.19 | 0.847 
ilr factor [1] × familyDoest not live with parents | [-0.14,  0.03] |   -1.31 | 0.189 
ilr factor [2] × familyDoest not live with parents | [-0.14,  0.03] |   -1.36 | 0.175 
ilr factor [3] × familyDoest not live with parents | [-0.07,  0.09] |    0.23 | 0.816 
ilr factor [1] × ses                               | [ 0.00,  0.00] |    0.16 | 0.871 
ilr factor [2] × ses                               | [ 0.00,  0.00] |    0.72 | 0.469 
ilr factor [3] × ses                               | [ 0.00,  0.00] |    0.28 | 0.778 
ilr factor [1] × survey2022 × age                  | [-0.01,  0.05] |    1.34 | 0.181 
ilr factor [2] × survey2022 × age                  | [-0.02,  0.04] |    0.69 | 0.488 
ilr factor [3] × survey2022 × age                  | [-0.03,  0.03] |    0.10 | 0.922 

# Random Effects

Parameter                 | Coefficient
---------------------------------------
SD (Intercept: id:campus) |        0.26
SD (Intercept: campus)    |        0.04
SD (Residual)             |        0.19
```

```
Parameter             |   Chi2 | df |      p
--------------------------------------------
ilr_factor            |  80.66 |  3 | < .001
ilr_factor:survey     |   1.95 |  3 | 0.583 
ilr_factor:sex        | 120.38 |  3 | < .001
ilr_factor:age        |   2.87 |  3 | 0.412 
ilr_factor:family     |  17.71 |  6 | 0.007 
ilr_factor:ses        |   0.71 |  3 | 0.872 
ilr_factor:survey:age |   2.87 |  3 | 0.411 

Anova Table (Type 3 tests)
```

```
[1] " "
[1] "family"
# Fixed Effects

Parameter                                                       | Coefficient
-----------------------------------------------------------------------------
ilr factor [1]                                                  |        1.32
ilr factor [2]                                                  |        1.40
ilr factor [3]                                                  |        0.90
ilr factor [1] × survey2022                                     |        0.08
ilr factor [2] × survey2022                                     |        0.07
ilr factor [3] × survey2022                                     |        0.04
ilr factor [1] × sexFemale                                      |        0.14
ilr factor [2] × sexFemale                                      |       -0.02
ilr factor [3] × sexFemale                                      |        0.06
ilr factor [1] × age                                            |        0.01
ilr factor [2] × age                                            |        0.01
ilr factor [3] × age                                            |   -7.62e-03
ilr factor [1] × familySingle parent                            |       -0.06
ilr factor [2] × familySingle parent                            |   -3.91e-03
ilr factor [3] × familySingle parent                            |    8.63e-05
ilr factor [1] × familyDoest not live with parents              |        0.05
ilr factor [2] × familyDoest not live with parents              |    9.33e-03
ilr factor [3] × familyDoest not live with parents              |        0.04
ilr factor [1] × ses                                            |    5.66e-05
ilr factor [2] × ses                                            |    6.21e-04
ilr factor [3] × ses                                            |    2.42e-04
ilr factor [1] × survey2022 × familySingle parent               |        0.02
ilr factor [2] × survey2022 × familySingle parent               |   -6.19e-03
ilr factor [3] × survey2022 × familySingle parent               |   -8.99e-03
ilr factor [1] × survey2022 × familyDoest not live with parents |       -0.22
ilr factor [2] × survey2022 × familyDoest not live with parents |       -0.14
ilr factor [3] × survey2022 × familyDoest not live with parents |       -0.07

Parameter                                                       |       SE
--------------------------------------------------------------------------
ilr factor [1]                                                  |     0.14
ilr factor [2]                                                  |     0.14
ilr factor [3]                                                  |     0.14
ilr factor [1] × survey2022                                     |     0.02
ilr factor [2] × survey2022                                     |     0.02
ilr factor [3] × survey2022                                     |     0.02
ilr factor [1] × sexFemale                                      |     0.02
ilr factor [2] × sexFemale                                      |     0.02
ilr factor [3] × sexFemale                                      |     0.02
ilr factor [1] × age                                            | 7.84e-03
ilr factor [2] × age                                            | 7.84e-03
ilr factor [3] × age                                            | 7.84e-03
ilr factor [1] × familySingle parent                            |     0.03
ilr factor [2] × familySingle parent                            |     0.03
ilr factor [3] × familySingle parent                            |     0.03
ilr factor [1] × familyDoest not live with parents              |     0.06
ilr factor [2] × familyDoest not live with parents              |     0.06
ilr factor [3] × familyDoest not live with parents              |     0.06
ilr factor [1] × ses                                            | 9.32e-04
ilr factor [2] × ses                                            | 9.32e-04
ilr factor [3] × ses                                            | 9.32e-04
ilr factor [1] × survey2022 × familySingle parent               |     0.04
ilr factor [2] × survey2022 × familySingle parent               |     0.04
ilr factor [3] × survey2022 × familySingle parent               |     0.04
ilr factor [1] × survey2022 × familyDoest not live with parents |     0.08
ilr factor [2] × survey2022 × familyDoest not live with parents |     0.08
ilr factor [3] × survey2022 × familyDoest not live with parents |     0.08

Parameter                                                      
---------------------------------------------------------------
ilr factor [1]                                                 
ilr factor [2]                                                 
ilr factor [3]                                                 
ilr factor [1] × survey2022                                    
ilr factor [2] × survey2022                                    
ilr factor [3] × survey2022                                    
ilr factor [1] × sexFemale                                     
ilr factor [2] × sexFemale                                     
ilr factor [3] × sexFemale                                     
ilr factor [1] × age                                           
ilr factor [2] × age                                           
ilr factor [3] × age                                           
ilr factor [1] × familySingle parent                           
ilr factor [2] × familySingle parent                           
ilr factor [3] × familySingle parent                           
ilr factor [1] × familyDoest not live with parents             
ilr factor [2] × familyDoest not live with parents             
ilr factor [3] × familyDoest not live with parents             
ilr factor [1] × ses                                           
ilr factor [2] × ses                                           
ilr factor [3] × ses                                           
ilr factor [1] × survey2022 × familySingle parent              
ilr factor [2] × survey2022 × familySingle parent              
ilr factor [3] × survey2022 × familySingle parent              
ilr factor [1] × survey2022 × familyDoest not live with parents
ilr factor [2] × survey2022 × familyDoest not live with parents
ilr factor [3] × survey2022 × familyDoest not live with parents

        95% CI |  t(3798) |      p
----------------------------------
[ 1.05,  1.59] |     9.63 | < .001
[ 1.13,  1.67] |    10.22 | < .001
[ 0.63,  1.16] |     6.53 | < .001
[ 0.04,  0.13] |     3.67 | < .001
[ 0.02,  0.11] |     2.85 | 0.004 
[ 0.00,  0.09] |     1.79 | 0.074 
[ 0.10,  0.17] |     7.53 | < .001
[-0.05,  0.02] |    -0.93 | 0.351 
[ 0.03,  0.10] |     3.36 | < .001
[ 0.00,  0.03] |     1.50 | 0.134 
[ 0.00,  0.03] |     1.76 | 0.078 
[-0.02,  0.01] |    -0.97 | 0.331 
[-0.12, -0.01] |    -2.36 | 0.018 
[-0.06,  0.05] |    -0.14 | 0.886 
[-0.05,  0.05] | 3.18e-03 | 0.997 
[-0.06,  0.16] |     0.87 | 0.385 
[-0.10,  0.12] |     0.17 | 0.866 
[-0.07,  0.15] |     0.75 | 0.456 
[ 0.00,  0.00] |     0.06 | 0.952 
[ 0.00,  0.00] |     0.67 | 0.505 
[ 0.00,  0.00] |     0.26 | 0.795 
[-0.06,  0.09] |     0.42 | 0.676 
[-0.08,  0.07] |    -0.16 | 0.872 
[-0.08,  0.07] |    -0.23 | 0.815 
[-0.38, -0.06] |    -2.72 | 0.007 
[-0.30,  0.02] |    -1.75 | 0.081 
[-0.23,  0.09] |    -0.86 | 0.391 

# Random Effects

Parameter                 | Coefficient
---------------------------------------
SD (Intercept: id:campus) |        0.26
SD (Intercept: campus)    |        0.04
SD (Residual)             |        0.19
```

```
Parameter                |   Chi2 | df |      p
-----------------------------------------------
ilr_factor               | 123.06 |  3 | < .001
ilr_factor:survey        |  15.13 |  3 | 0.002 
ilr_factor:sex           | 119.73 |  3 | < .001
ilr_factor:age           |  13.89 |  3 | 0.003 
ilr_factor:family        |  13.48 |  6 | 0.036 
ilr_factor:ses           |   0.69 |  3 | 0.875 
ilr_factor:survey:family |  10.68 |  6 | 0.099 

Anova Table (Type 3 tests)
```

```
[1] " "
[1] "ses"
# Fixed Effects

Parameter                                          | Coefficient |       SE
---------------------------------------------------------------------------
ilr factor [1]                                     |        1.39 |     0.14
ilr factor [2]                                     |        1.44 |     0.14
ilr factor [3]                                     |        0.90 |     0.14
ilr factor [1] × survey2022                        |   -7.61e-03 |     0.07
ilr factor [2] × survey2022                        |        0.01 |     0.07
ilr factor [3] × survey2022                        |        0.04 |     0.07
ilr factor [1] × sexFemale                         |        0.13 |     0.02
ilr factor [2] × sexFemale                         |       -0.02 |     0.02
ilr factor [3] × sexFemale                         |        0.06 |     0.02
ilr factor [1] × age                               |    9.82e-03 | 7.82e-03
ilr factor [2] × age                               |        0.01 | 7.82e-03
ilr factor [3] × age                               |   -7.99e-03 | 7.82e-03
ilr factor [1] × familySingle parent               |       -0.06 |     0.02
ilr factor [2] × familySingle parent               |   -6.24e-03 |     0.02
ilr factor [3] × familySingle parent               |   -3.91e-03 |     0.02
ilr factor [1] × familyDoest not live with parents |       -0.05 |     0.04
ilr factor [2] × familyDoest not live with parents |       -0.05 |     0.04
ilr factor [3] × familyDoest not live with parents |    9.58e-03 |     0.04
ilr factor [1] × ses                               |   -9.17e-04 | 1.27e-03
ilr factor [2] × ses                               |    1.13e-04 | 1.27e-03
ilr factor [3] × ses                               |    3.94e-04 | 1.27e-03
ilr factor [1] × survey2022 × ses                  |    2.22e-03 | 1.79e-03
ilr factor [2] × survey2022 × ses                  |    1.17e-03 | 1.79e-03
ilr factor [3] × survey2022 × ses                  |   -2.59e-04 | 1.79e-03

Parameter                                          |         95% CI | t(3801) |      p
--------------------------------------------------------------------------------------
ilr factor [1]                                     | [ 1.12,  1.67] |    9.84 | < .001
ilr factor [2]                                     | [ 1.16,  1.72] |   10.18 | < .001
ilr factor [3]                                     | [ 0.62,  1.18] |    6.34 | < .001
ilr factor [1] × survey2022                        | [-0.15,  0.13] |   -0.11 | 0.916 
ilr factor [2] × survey2022                        | [-0.13,  0.15] |    0.15 | 0.882 
ilr factor [3] × survey2022                        | [-0.10,  0.19] |    0.62 | 0.534 
ilr factor [1] × sexFemale                         | [ 0.10,  0.17] |    7.45 | < .001
ilr factor [2] × sexFemale                         | [-0.05,  0.02] |   -0.99 | 0.325 
ilr factor [3] × sexFemale                         | [ 0.03,  0.10] |    3.34 | < .001
ilr factor [1] × age                               | [-0.01,  0.03] |    1.26 | 0.209 
ilr factor [2] × age                               | [ 0.00,  0.03] |    1.64 | 0.101 
ilr factor [3] × age                               | [-0.02,  0.01] |   -1.02 | 0.307 
ilr factor [1] × familySingle parent               | [-0.09, -0.02] |   -2.80 | 0.005 
ilr factor [2] × familySingle parent               | [-0.04,  0.03] |   -0.32 | 0.752 
ilr factor [3] × familySingle parent               | [-0.04,  0.03] |   -0.20 | 0.843 
ilr factor [1] × familyDoest not live with parents | [-0.13,  0.03] |   -1.21 | 0.228 
ilr factor [2] × familyDoest not live with parents | [-0.13,  0.03] |   -1.30 | 0.193 
ilr factor [3] × familyDoest not live with parents | [-0.07,  0.09] |    0.23 | 0.817 
ilr factor [1] × ses                               | [ 0.00,  0.00] |   -0.72 | 0.472 
ilr factor [2] × ses                               | [ 0.00,  0.00] |    0.09 | 0.929 
ilr factor [3] × ses                               | [ 0.00,  0.00] |    0.31 | 0.757 
ilr factor [1] × survey2022 × ses                  | [ 0.00,  0.01] |    1.24 | 0.214 
ilr factor [2] × survey2022 × ses                  | [ 0.00,  0.00] |    0.66 | 0.512 
ilr factor [3] × survey2022 × ses                  | [ 0.00,  0.00] |   -0.14 | 0.885 

# Random Effects

Parameter                 | Coefficient
---------------------------------------
SD (Intercept: id:campus) |        0.26
SD (Intercept: campus)    |        0.04
SD (Residual)             |        0.19
```

```
Parameter             |   Chi2 | df |      p
--------------------------------------------
ilr_factor            | 126.31 |  3 | < .001
ilr_factor:survey     |   0.85 |  3 | 0.838 
ilr_factor:sex        | 118.65 |  3 | < .001
ilr_factor:age        |  12.44 |  3 | 0.006 
ilr_factor:family     |  17.84 |  6 | 0.007 
ilr_factor:ses        |   1.71 |  3 | 0.634 
ilr_factor:survey:ses |   3.24 |  3 | 0.356 

Anova Table (Type 3 tests)
```

## 1.7 Summary (Table 5.1)

Parameters of interest from the models

```
tbl3 <- bind_rows(inter_sex, inter_age, inter_family, inter_ses) %>% 
  mutate(
    p = as.character(p),
    p = case_match(
      p,
      '0.000' ~ '<0.001',
      .default = p))

tbl3 %>% 
  kbl(align = 'l',
      caption = 'Interaction effect between each moderator and survey for the composition') %>% 
  kable_styling(bootstrap_options = c("striped", "hover", "condensed", "responsive"))
```

Interaction effect between each moderator and survey for the composition

| parameter | df | chi2 | p |
| --- | --- | --- | --- |
| ilr\_factor \* survey \* sex | 3 | 2.260950 | 0.520 |
| ilr\_factor \* survey \* age | 3 | 2.874033 | 0.411 |
| ilr\_factor \* survey \* family | 6 | 10.676561 | 0.099 |
| ilr\_factor \* survey \* ses | 3 | 3.237350 | 0.356 |

# 2 Prospective data

## 2.1 Visualization of the compositional data

The variables MVPA, LIPA, SB and SPT are declared as a single compositional vector using the `compositional::acomp()` function.

```
labels <- c("MVPA","LIPA","SB","SPT")

coda <- Data_long %>%
  mutate(
    activity = cbind(MVPA, LIPA, SB, SPT),
    composition = acomp(activity))
```

Compositional outliers based on Robust Mahalanobis Distance are then inspected.

```
outlierplot.acomp(coda$composition, type = 'scatter', robust = T)
```

```
outlierplot.acomp(coda$composition, type = 'biplot', robust = T)
```

```
outliers <- outlierplot.acomp(coda$composition, type = 'distdist', robust = T)
```

```
outliers <-  data.frame(out_class = outliers[["classes"]],
                        out_mahal = outliers[["NormalMahalanobisDist"]],
                        out_mahal_rob = outliers[["RobustMahalanobisDist"]],
                        out_mahal_crit = outliers[["crit"]])


coda <- bind_cols(coda, outliers) 
coda %>% filter(out_class!='ok') %>% 
  select(MVPA, LIPA, SB, SPT, out_mahal_rob, out_mahal) %>% 
  kbl() %>% kable_styling(bootstrap_options = c("striped", "condensed"), full_width = F)
```

| MVPA | LIPA | SB | SPT | out\_mahal\_rob | out\_mahal |
| --- | --- | --- | --- | --- | --- |
| 19.176 | 559.688 | 379.327 | 481.810 | 5.389688 | 4.670840 |
| 67.387 | 468.595 | 379.265 | 524.753 | 4.725448 | 3.917231 |
| 2.494 | 128.759 | 847.062 | 461.685 | 4.971526 | 4.325596 |

Compositional means expressed in proportion relative to the 24 hours

```
coda %>% group_by(survey) %>% 
  reframe(
    avg = mean.acomp(composition),
    beh = labels) %>% 
  ungroup() %>% 
  mutate(avg=round(avg*100,2)) %>% 
  pivot_wider(
    names_from = c(beh),
    values_from = c(avg)
  ) %>% 
  kbl() %>% kable_styling(bootstrap_options = c("striped", "condensed"), full_width = F)
```

| survey | MVPA | LIPA | SB | SPT |
| --- | --- | --- | --- | --- |
| 2019 | 2.21 | 19.54 | 46.4 | 31.85 |
| 2022 | 1.78 | 18.41 | 48.7 | 31.10 |

```
# Variation matrix

var1 <- coda %>% 
  filter(survey=='2019') %>% 
  select(composition) %>% 
  reframe(variation = round(
    compositions::variation(composition), 3)) %>% 
  as.matrix(.) %>% data.frame() %>% 
  mutate(Variables = labels) %>% 
  relocate(Variables)

names(var1) <- c('Variables',labels)
var1 %>% kbl(caption = 'Variation matrix for 2019 data') %>% kable_styling(bootstrap_options = c("striped", "condensed"))
```

Variation matrix for 2019 data

| Variables | MVPA | LIPA | SB | SPT |
| --- | --- | --- | --- | --- |
| MVPA | 0.000 | 0.269 | 0.371 | 0.322 |
| LIPA | 0.269 | 0.000 | 0.103 | 0.066 |
| SB | 0.371 | 0.103 | 0.000 | 0.039 |
| SPT | 0.322 | 0.066 | 0.039 | 0.000 |

```
var2 <- coda %>% 
  filter(survey=='2022') %>% 
  select(composition) %>% 
  reframe(variation = round(
    compositions::variation(composition), 3)) %>% 
  as.matrix(.) %>% data.frame() %>% 
  mutate(Variables = labels) %>% 
  relocate(Variables)

names(var2) <- c('Variables',labels)
var2 %>% kbl(caption = 'Variation matrix for 2022 data') %>% kable_styling(bootstrap_options = c("striped", "condensed"))
```

Variation matrix for 2022 data

| Variables | MVPA | LIPA | SB | SPT |
| --- | --- | --- | --- | --- |
| MVPA | 0.000 | 0.300 | 0.461 | 0.411 |
| LIPA | 0.300 | 0.000 | 0.133 | 0.099 |
| SB | 0.461 | 0.133 | 0.000 | 0.039 |
| SPT | 0.411 | 0.099 | 0.039 | 0.000 |

3D Ternary diagram

```
compositions::plot3D(coda$composition[,1:4], cex=5 ,
                     color=coda$survey, alpha=0.6, axis.col = 'black') 
rglwidget()
```

## 2.2 Preparing data for modeling

```
# defining SBP

# creating pivot coordinates
ilr_mvpa <-  coda %>% 
  select(all_of(labels)) %>% 
  data.matrix() %>% 
  pivotCoord(1) %>% 
  data.frame()
names(ilr_mvpa) <- c('ilr_mvpa.1', 'ilr_mvpa.2', 'ilr_mvpa.3')

ilr_lipa <-  coda %>% 
  select(all_of(labels)) %>% 
  data.matrix() %>% 
  pivotCoord(2) %>% 
  data.frame()
names(ilr_lipa) <- c('ilr_lipa.1', 'ilr_lipa.2', 'ilr_lipa.3')

ilr_sb <-  coda %>% 
  select(all_of(labels)) %>% 
  data.matrix() %>% 
  pivotCoord(3) %>% 
  data.frame()
names(ilr_sb) <- c('ilr_sb.1', 'ilr_sb.2', 'ilr_sb.3')

ilr_spt <-  coda %>% 
  select(all_of(labels)) %>% 
  data.matrix() %>% 
  pivotCoord(4) %>% 
  data.frame()
names(ilr_spt) <- c('ilr_spt.1', 'ilr_spt.2', 'ilr_spt.3')

ilr_simplex <-  coda %>% 
  select(all_of(labels)) %>% 
  ilr() %>% data.frame()

coda <- bind_cols(coda, ilr_mvpa, ilr_lipa, ilr_sb, ilr_spt, ilr_simplex) %>% 
  mutate(
    ilr.1 = V1, 
    ilr.2 = V2, 
    ilr.3 = V3
  )

coda_stacked <- coda %>% 
  pivot_longer(cols = starts_with(
    c("ilr.", "ilr_mvpa.", "ilr_lipa.", "ilr_sb.", "ilr_spt.")), 
    names_to = c(".value", "ilr_factor"),
    names_sep = "\\.") %>% 
  mutate(ilr_factor = as_factor(ilr_factor))
```

## 2.3 Overall composition difference between time points

The `lmer` function was used to fit the multilevel model applied to the stacked data. This approach was previously used for modeling compositional data (see (Lim et al. 2023)) and instructions are provided elsewhere (see (Rosen 2023)). The `emmeans` function is used to get adjusted predictions. The `parameters` function is used to extract specific parameters of interest. The stored parameters are used to create the summary table presented in the paper (Section 2.5).

```
model <- lmer(ilr ~  -1 +
                ilr_factor +
                ilr_factor:survey +
                ilr_factor:sex +
                ilr_factor:family +
                ilr_factor:ses +
                ilr_factor:age +
                (0 + ilr_factor | campus/id), 
              coda_stacked,
              control = lmerControl(
                optimizer ='optimx', optCtrl=list(method='L-BFGS-B'),
                check.conv.singular = 
                  .makeCC(action = "ignore", 
                          tol = formals(isSingular)$tol)))

check_model(model,  check = c("pp_check", "linearity", "homogeneity", "outliers"))
```

```
check_model(model,  check = c("qq", "normality", "reqq"))
```

```
parameters(model)
```

```
# Fixed Effects

Parameter                                          | Coefficient |       SE
---------------------------------------------------------------------------
ilr factor [1]                                     |        1.90 |     0.44
ilr factor [2]                                     |        1.99 |     0.42
ilr factor [3]                                     |        1.20 |     0.29
ilr factor [1] × survey2022                        |        0.10 |     0.02
ilr factor [2] × survey2022                        |        0.15 |     0.02
ilr factor [3] × survey2022                        |        0.04 |     0.02
ilr factor [1] × sexFemale                         |        0.17 |     0.04
ilr factor [2] × sexFemale                         |       -0.02 |     0.04
ilr factor [3] × sexFemale                         |        0.06 |     0.03
ilr factor [1] × familySingle parent               |       -0.04 |     0.04
ilr factor [2] × familySingle parent               |        0.03 |     0.04
ilr factor [3] × familySingle parent               |    7.02e-03 |     0.03
ilr factor [1] × familyDoest not live with parents |        0.10 |     0.09
ilr factor [2] × familyDoest not live with parents |        0.04 |     0.09
ilr factor [3] × familyDoest not live with parents |        0.05 |     0.06
ilr factor [1] × ses                               |   -1.52e-03 | 2.36e-03
ilr factor [2] × ses                               |    3.91e-04 | 2.28e-03
ilr factor [3] × ses                               |    1.00e-03 | 1.57e-03
ilr factor [1] × age                               |       -0.02 |     0.03
ilr factor [2] × age                               |       -0.02 |     0.03
ilr factor [3] × age                               |       -0.03 |     0.02

Parameter                                          |        95% CI | t(1127) |      p
-------------------------------------------------------------------------------------
ilr factor [1]                                     | [ 1.05, 2.76] |    4.37 | < .001
ilr factor [2]                                     | [ 1.16, 2.82] |    4.72 | < .001
ilr factor [3]                                     | [ 0.62, 1.78] |    4.08 | < .001
ilr factor [1] × survey2022                        | [ 0.05, 0.14] |    4.33 | < .001
ilr factor [2] × survey2022                        | [ 0.10, 0.19] |    6.58 | < .001
ilr factor [3] × survey2022                        | [ 0.00, 0.08] |    1.74 | 0.083 
ilr factor [1] × sexFemale                         | [ 0.08, 0.25] |    3.81 | < .001
ilr factor [2] × sexFemale                         | [-0.10, 0.07] |   -0.41 | 0.680 
ilr factor [3] × sexFemale                         | [ 0.00, 0.12] |    2.03 | 0.042 
ilr factor [1] × familySingle parent               | [-0.13, 0.04] |   -0.95 | 0.344 
ilr factor [2] × familySingle parent               | [-0.05, 0.11] |    0.72 | 0.470 
ilr factor [3] × familySingle parent               | [-0.05, 0.07] |    0.23 | 0.816 
ilr factor [1] × familyDoest not live with parents | [-0.08, 0.28] |    1.09 | 0.278 
ilr factor [2] × familyDoest not live with parents | [-0.14, 0.21] |    0.44 | 0.661 
ilr factor [3] × familyDoest not live with parents | [-0.08, 0.18] |    0.78 | 0.436 
ilr factor [1] × ses                               | [-0.01, 0.00] |   -0.64 | 0.520 
ilr factor [2] × ses                               | [ 0.00, 0.00] |    0.17 | 0.864 
ilr factor [3] × ses                               | [ 0.00, 0.00] |    0.64 | 0.523 
ilr factor [1] × age                               | [-0.08, 0.03] |   -0.89 | 0.376 
ilr factor [2] × age                               | [-0.08, 0.03] |   -0.97 | 0.333 
ilr factor [3] × age                               | [-0.06, 0.01] |   -1.64 | 0.101 

# Random Effects

Parameter                                | Coefficient
------------------------------------------------------
SD (ilr_factor1: id:campus)              |        0.29
SD (ilr_factor2: id:campus)              |        0.28
SD (ilr_factor3: id:campus)              |        0.16
SD (ilr_factor1: campus)                 |        0.07
SD (ilr_factor2: campus)                 |        0.07
SD (ilr_factor3: campus)                 |        0.05
Cor (ilr_factor1~ilr_factor2: id:campus) |        0.85
Cor (ilr_factor1~ilr_factor3: id:campus) |        0.90
Cor (ilr_factor2~ilr_factor3: id:campus) |        0.99
Cor (ilr_factor1~ilr_factor2: campus)    |        0.70
Cor (ilr_factor1~ilr_factor3: campus)    |        0.93
Cor (ilr_factor2~ilr_factor3: campus)    |        0.91
SD (Residual)                            |        0.19
```

```
parameters(car::Anova(model, type = "III"))
```

```
Parameter         |  Chi2 | df |      p
---------------------------------------
ilr_factor        | 26.62 |  3 | < .001
ilr_factor:survey | 60.44 |  3 | < .001
ilr_factor:sex    | 31.05 |  3 | < .001
ilr_factor:family |  5.49 |  6 | 0.483 
ilr_factor:ses    |  1.98 |  3 | 0.577 
ilr_factor:age    |  2.69 |  3 | 0.442 

Anova Table (Type 3 tests)
```

```
comp <- parameters(car::Anova(model, type = "III")) %>% 
  filter(Parameter=='ilr_factor:survey') %>% 
  select(p) %>% 
  mutate(
    parameter = "Composition",
    p = sprintf(p, fmt = "%.3f")) %>% 
  clean_names() %>% 
  as_tibble()

pred <- tidy(emmeans::emmeans(model, ~ ilr_factor*survey, weights = "proportional")) %>%
  select(c("estimate", 'std.error', 'survey', 'ilr_factor')) %>% 
  pivot_wider(names_from = 'ilr_factor', values_from =c("estimate", "std.error")) %>% 
  # Change to include CI
  select(survey, c(starts_with('estimate'))) %>% 
  mutate(geo = clo(ilrInv(cbind(
    `estimate_1`, 
    `estimate_2`, 
    `estimate_3`)), total=1440),
    geo = round(geo, 1))

pred <- data.frame(pred$geo,
                   survey = pred$survey) %>% 
  relocate(survey)

colnames(pred)[2:5] <- labels 

pred <- transpose(pred, make.names = 1)
pred <- rbind(NA, pred)
```

## 2.4 Pivot coordinates differences between time points

The second set of models were fitted using the pivot coordinates as dependent variables. The `ilr_factor1:survey` coefficient is interpreted as the differences between surveys for the relative contribution of the numerator to the remaining behaviors.

```
outcomes <- c('ilr_mvpa', 'ilr_lipa', 'ilr_sb', 'ilr_spt')

# Running the models and saving parameters
for (outcome in outcomes) {
  
  model <- lmer(get(outcome) ~ -1 +
                  ilr_factor +
                  ilr_factor:survey +
                  ilr_factor:age +
                  ilr_factor:sex +
                  ilr_factor:family +
                  ilr_factor:ses +
                  (0 + ilr_factor | campus/id), 
                coda_stacked,
                control = lmerControl(
                  optimizer ='optimx', optCtrl=list(method='L-BFGS-B'),
                  check.conv.singular = 
                    .makeCC(action = "ignore", 
                            tol = formals(isSingular)$tol)))
  print(' ')
  print(outcome)
  parameters(model) %>% print()
  
  coef <- parameters(model) %>% 
    filter(Parameter=='ilr_factor1:survey2022') %>% 
    mutate(
      Coefficient = sprintf(Coefficient, fmt = "%.3f"),
      p = sprintf(p, fmt = "%.3f")) %>% 
    clean_names() %>% 
    select(c('parameter', 'coefficient', 'p')) %>% 
    as_tibble() 
  
  assign(paste0('m1_', outcome), model)
  assign(paste0('c1_', outcome), coef)
  
  remove(list = c('model'))
}
```

```
[1] " "
[1] "ilr_mvpa"
# Fixed Effects

Parameter                                          | Coefficient |       SE
---------------------------------------------------------------------------
ilr factor [1]                                     |       -2.89 |     0.57
ilr factor [2]                                     |       -0.79 |     0.28
ilr factor [3]                                     |        0.17 |     0.21
ilr factor [1] × survey2022                        |       -0.16 |     0.02
ilr factor [2] × survey2022                        |       -0.06 |     0.02
ilr factor [3] × survey2022                        |        0.05 |     0.02
ilr factor [1] × age                               |        0.04 |     0.03
ilr factor [2] × age                               |        0.02 |     0.02
ilr factor [3] × age                               |    9.62e-03 |     0.01
ilr factor [1] × sexFemale                         |       -0.15 |     0.06
ilr factor [2] × sexFemale                         |        0.08 |     0.03
ilr factor [3] × sexFemale                         |       -0.06 |     0.02
ilr factor [1] × familySingle parent               |        0.02 |     0.06
ilr factor [2] × familySingle parent               |       -0.05 |     0.03
ilr factor [3] × familySingle parent               |        0.01 |     0.02
ilr factor [1] × familyDoest not live with parents |       -0.12 |     0.12
ilr factor [2] × familyDoest not live with parents |    8.08e-03 |     0.06
ilr factor [3] × familyDoest not live with parents |       -0.02 |     0.05
ilr factor [1] × ses                               |    7.22e-04 | 3.11e-03
ilr factor [2] × ses                               |   -1.61e-03 | 1.51e-03
ilr factor [3] × ses                               |   -5.93e-04 | 1.14e-03

Parameter                                          |         95% CI | t(1127) |      p
--------------------------------------------------------------------------------------
ilr factor [1]                                     | [-4.02, -1.77] |   -5.05 | < .001
ilr factor [2]                                     | [-1.35, -0.24] |   -2.80 | 0.005 
ilr factor [3]                                     | [-0.25,  0.59] |    0.78 | 0.434 
ilr factor [1] × survey2022                        | [-0.20, -0.11] |   -6.93 | < .001
ilr factor [2] × survey2022                        | [-0.10, -0.02] |   -2.71 | 0.007 
ilr factor [3] × survey2022                        | [ 0.01,  0.09] |    2.58 | 0.010 
ilr factor [1] × age                               | [-0.03,  0.11] |    1.17 | 0.243 
ilr factor [2] × age                               | [-0.02,  0.05] |    0.98 | 0.329 
ilr factor [3] × age                               | [-0.02,  0.04] |    0.73 | 0.463 
ilr factor [1] × sexFemale                         | [-0.26, -0.03] |   -2.56 | 0.011 
ilr factor [2] × sexFemale                         | [ 0.03,  0.14] |    2.86 | 0.004 
ilr factor [3] × sexFemale                         | [-0.10, -0.02] |   -2.81 | 0.005 
ilr factor [1] × familySingle parent               | [-0.09,  0.13] |    0.30 | 0.762 
ilr factor [2] × familySingle parent               | [-0.11,  0.01] |   -1.60 | 0.110 
ilr factor [3] × familySingle parent               | [-0.03,  0.06] |    0.52 | 0.606 
ilr factor [1] × familyDoest not live with parents | [-0.35,  0.11] |   -1.00 | 0.320 
ilr factor [2] × familyDoest not live with parents | [-0.12,  0.14] |    0.12 | 0.901 
ilr factor [3] × familyDoest not live with parents | [-0.12,  0.08] |   -0.36 | 0.716 
ilr factor [1] × ses                               | [-0.01,  0.01] |    0.23 | 0.816 
ilr factor [2] × ses                               | [ 0.00,  0.00] |   -1.07 | 0.286 
ilr factor [3] × ses                               | [ 0.00,  0.00] |   -0.52 | 0.602 

# Random Effects

Parameter                                | Coefficient
------------------------------------------------------
SD (ilr_factor1: id:campus)              |        0.41
SD (ilr_factor2: id:campus)              |        0.14
SD (ilr_factor3: id:campus)              |        0.04
SD (ilr_factor1: campus)                 |        0.10
SD (ilr_factor2: campus)                 |        0.05
SD (ilr_factor3: campus)                 |        0.02
Cor (ilr_factor1~ilr_factor2: id:campus) |        0.59
Cor (ilr_factor1~ilr_factor3: id:campus) |       -0.75
Cor (ilr_factor2~ilr_factor3: id:campus) |       -0.98
Cor (ilr_factor1~ilr_factor2: campus)    |        0.62
Cor (ilr_factor1~ilr_factor3: campus)    |        0.22
Cor (ilr_factor2~ilr_factor3: campus)    |       -0.63
SD (Residual)                            |        0.19
```

```
[1] " "
[1] "ilr_lipa"
# Fixed Effects

Parameter                                          | Coefficient |       SE
---------------------------------------------------------------------------
ilr factor [1]                                     |        0.22 |     0.27
ilr factor [2]                                     |       -2.99 |     0.58
ilr factor [3]                                     |        0.17 |     0.21
ilr factor [1] × survey2022                        |   -2.10e-03 |     0.02
ilr factor [2] × survey2022                        |       -0.17 |     0.02
ilr factor [3] × survey2022                        |        0.05 |     0.02
ilr factor [1] × age                               |    2.32e-03 |     0.02
ilr factor [2] × age                               |        0.04 |     0.04
ilr factor [3] × age                               |    9.62e-03 |     0.01
ilr factor [1] × sexFemale                         |        0.12 |     0.03
ilr factor [2] × sexFemale                         |       -0.11 |     0.06
ilr factor [3] × sexFemale                         |       -0.06 |     0.02
ilr factor [1] × familySingle parent               |       -0.05 |     0.03
ilr factor [2] × familySingle parent               |    2.51e-04 |     0.06
ilr factor [3] × familySingle parent               |        0.01 |     0.02
ilr factor [1] × familyDoest not live with parents |        0.05 |     0.06
ilr factor [2] × familyDoest not live with parents |       -0.11 |     0.12
ilr factor [3] × familyDoest not live with parents |       -0.02 |     0.05
ilr factor [1] × ses                               |   -1.76e-03 | 1.42e-03
ilr factor [2] × ses                               |    1.44e-04 | 3.15e-03
ilr factor [3] × ses                               |   -5.93e-04 | 1.14e-03

Parameter                                          |         95% CI |  t(1127) |      p
---------------------------------------------------------------------------------------
ilr factor [1]                                     | [-0.31,  0.74] |     0.81 | 0.420 
ilr factor [2]                                     | [-4.13, -1.85] |    -5.15 | < .001
ilr factor [3]                                     | [-0.25,  0.59] |     0.78 | 0.434 
ilr factor [1] × survey2022                        | [-0.04,  0.04] |    -0.10 | 0.923 
ilr factor [2] × survey2022                        | [-0.22, -0.12] |    -7.37 | < .001
ilr factor [3] × survey2022                        | [ 0.01,  0.09] |     2.58 | 0.010 
ilr factor [1] × age                               | [-0.03,  0.03] |     0.14 | 0.887 
ilr factor [2] × age                               | [-0.03,  0.11] |     1.25 | 0.213 
ilr factor [3] × age                               | [-0.02,  0.04] |     0.73 | 0.463 
ilr factor [1] × sexFemale                         | [ 0.07,  0.18] |     4.75 | < .001
ilr factor [2] × sexFemale                         | [-0.23,  0.00] |    -1.92 | 0.055 
ilr factor [3] × sexFemale                         | [-0.10, -0.02] |    -2.81 | 0.005 
ilr factor [1] × familySingle parent               | [-0.11,  0.01] |    -1.78 | 0.076 
ilr factor [2] × familySingle parent               | [-0.11,  0.11] | 4.38e-03 | 0.997 
ilr factor [3] × familySingle parent               | [-0.03,  0.06] |     0.52 | 0.606 
ilr factor [1] × familyDoest not live with parents | [-0.08,  0.17] |     0.75 | 0.454 
ilr factor [2] × familyDoest not live with parents | [-0.34,  0.13] |    -0.91 | 0.366 
ilr factor [3] × familyDoest not live with parents | [-0.12,  0.08] |    -0.36 | 0.716 
ilr factor [1] × ses                               | [ 0.00,  0.00] |    -1.24 | 0.216 
ilr factor [2] × ses                               | [-0.01,  0.01] |     0.05 | 0.964 
ilr factor [3] × ses                               | [ 0.00,  0.00] |    -0.52 | 0.602 

# Random Effects

Parameter                                | Coefficient
------------------------------------------------------
SD (ilr_factor1: id:campus)              |        0.12
SD (ilr_factor2: id:campus)              |        0.42
SD (ilr_factor3: id:campus)              |        0.04
SD (ilr_factor1: campus)                 |        0.04
SD (ilr_factor2: campus)                 |        0.11
SD (ilr_factor3: campus)                 |        0.02
Cor (ilr_factor1~ilr_factor2: id:campus) |       -0.39
Cor (ilr_factor1~ilr_factor3: id:campus) |       -0.23
Cor (ilr_factor2~ilr_factor3: id:campus) |       -0.81
Cor (ilr_factor1~ilr_factor2: campus)    |       -0.07
Cor (ilr_factor1~ilr_factor3: campus)    |       -1.00
Cor (ilr_factor2~ilr_factor3: campus)    |        0.11
SD (Residual)                            |        0.19
```

```
[1] " "
[1] "ilr_sb"
# Fixed Effects

Parameter                                          | Coefficient |       SE
---------------------------------------------------------------------------
ilr factor [1]                                     |        1.48 |     0.35
ilr factor [2]                                     |       -2.55 |     0.52
ilr factor [3]                                     |       -0.60 |     0.25
ilr factor [1] × survey2022                        |        0.12 |     0.02
ilr factor [2] × survey2022                        |       -0.13 |     0.02
ilr factor [3] × survey2022                        |       -0.02 |     0.02
ilr factor [1] × age                               |       -0.01 |     0.02
ilr factor [2] × age                               |        0.04 |     0.03
ilr factor [3] × age                               |        0.02 |     0.02
ilr factor [1] × sexFemale                         |       -0.04 |     0.03
ilr factor [2] × sexFemale                         |       -0.17 |     0.05
ilr factor [3] × sexFemale                         |        0.04 |     0.02
ilr factor [1] × familySingle parent               |        0.03 |     0.04
ilr factor [2] × familySingle parent               |        0.03 |     0.05
ilr factor [3] × familySingle parent               |       -0.04 |     0.03
ilr factor [1] × familyDoest not live with parents |        0.02 |     0.07
ilr factor [2] × familyDoest not live with parents |       -0.12 |     0.11
ilr factor [3] × familyDoest not live with parents |   -2.23e-03 |     0.06
ilr factor [1] × ses                               |    3.49e-05 | 1.87e-03
ilr factor [2] × ses                               |    7.78e-04 | 2.82e-03
ilr factor [3] × ses                               |   -1.69e-03 | 1.34e-03

Parameter                                          |         95% CI | t(1127) |      p
--------------------------------------------------------------------------------------
ilr factor [1]                                     | [ 0.80,  2.16] |    4.25 | < .001
ilr factor [2]                                     | [-3.57, -1.53] |   -4.90 | < .001
ilr factor [3]                                     | [-1.10, -0.11] |   -2.39 | 0.017 
ilr factor [1] × survey2022                        | [ 0.08,  0.17] |    5.77 | < .001
ilr factor [2] × survey2022                        | [-0.17, -0.08] |   -5.52 | < .001
ilr factor [3] × survey2022                        | [-0.07,  0.02] |   -1.12 | 0.263 
ilr factor [1] × age                               | [-0.06,  0.03] |   -0.65 | 0.518 
ilr factor [2] × age                               | [-0.02,  0.10] |    1.21 | 0.226 
ilr factor [3] × age                               | [-0.01,  0.05] |    1.26 | 0.207 
ilr factor [1] × sexFemale                         | [-0.10,  0.03] |   -1.05 | 0.296 
ilr factor [2] × sexFemale                         | [-0.27, -0.07] |   -3.24 | 0.001 
ilr factor [3] × sexFemale                         | [-0.01,  0.09] |    1.62 | 0.105 
ilr factor [1] × familySingle parent               | [-0.04,  0.10] |    0.75 | 0.453 
ilr factor [2] × familySingle parent               | [-0.07,  0.13] |    0.53 | 0.593 
ilr factor [3] × familySingle parent               | [-0.09,  0.02] |   -1.32 | 0.188 
ilr factor [1] × familyDoest not live with parents | [-0.13,  0.17] |    0.27 | 0.789 
ilr factor [2] × familyDoest not live with parents | [-0.33,  0.09] |   -1.09 | 0.276 
ilr factor [3] × familyDoest not live with parents | [-0.12,  0.11] |   -0.04 | 0.970 
ilr factor [1] × ses                               | [ 0.00,  0.00] |    0.02 | 0.985 
ilr factor [2] × ses                               | [ 0.00,  0.01] |    0.28 | 0.782 
ilr factor [3] × ses                               | [ 0.00,  0.00] |   -1.26 | 0.207 

# Random Effects

Parameter                                | Coefficient
------------------------------------------------------
SD (ilr_factor1: id:campus)              |        0.21
SD (ilr_factor2: id:campus)              |        0.37
SD (ilr_factor3: id:campus)              |        0.10
SD (ilr_factor1: campus)                 |        0.05
SD (ilr_factor2: campus)                 |        0.09
SD (ilr_factor3: campus)                 |        0.04
Cor (ilr_factor1~ilr_factor2: id:campus) |       -0.91
Cor (ilr_factor1~ilr_factor3: id:campus) |       -0.81
Cor (ilr_factor2~ilr_factor3: id:campus) |        0.50
Cor (ilr_factor1~ilr_factor2: campus)    |       -0.74
Cor (ilr_factor1~ilr_factor3: campus)    |       -1.00
Cor (ilr_factor2~ilr_factor3: campus)    |        0.67
SD (Residual)                            |        0.19
```

```
[1] " "
[1] "ilr_spt"
# Fixed Effects

Parameter                                          | Coefficient |       SE
---------------------------------------------------------------------------
ilr factor [1]                                     |        1.20 |     0.29
ilr factor [2]                                     |       -2.63 |     0.54
ilr factor [3]                                     |       -0.77 |     0.28
ilr factor [1] × survey2022                        |        0.04 |     0.02
ilr factor [2] × survey2022                        |       -0.16 |     0.02
ilr factor [3] × survey2022                        |       -0.08 |     0.02
ilr factor [1] × age                               |       -0.03 |     0.02
ilr factor [2] × age                               |        0.03 |     0.03
ilr factor [3] × age                               |    9.80e-03 |     0.02
ilr factor [1] × sexFemale                         |        0.06 |     0.03
ilr factor [2] × sexFemale                         |       -0.14 |     0.05
ilr factor [3] × sexFemale                         |        0.10 |     0.03
ilr factor [1] × familySingle parent               |    6.99e-03 |     0.03
ilr factor [2] × familySingle parent               |        0.02 |     0.05
ilr factor [3] × familySingle parent               |       -0.05 |     0.03
ilr factor [1] × familyDoest not live with parents |        0.05 |     0.06
ilr factor [2] × familyDoest not live with parents |       -0.11 |     0.11
ilr factor [3] × familyDoest not live with parents |        0.02 |     0.06
ilr factor [1] × ses                               |    1.00e-03 | 1.57e-03
ilr factor [2] × ses                               |    1.12e-03 | 2.92e-03
ilr factor [3] × ses                               |   -1.10e-03 | 1.50e-03

Parameter                                          |         95% CI | t(1127) |      p
--------------------------------------------------------------------------------------
ilr factor [1]                                     | [ 0.63,  1.78] |    4.09 | < .001
ilr factor [2]                                     | [-3.69, -1.58] |   -4.91 | < .001
ilr factor [3]                                     | [-1.33, -0.22] |   -2.73 | 0.006 
ilr factor [1] × survey2022                        | [ 0.00,  0.08] |    1.73 | 0.084 
ilr factor [2] × survey2022                        | [-0.20, -0.11] |   -6.87 | < .001
ilr factor [3] × survey2022                        | [-0.12, -0.04] |   -3.60 | < .001
ilr factor [1] × age                               | [-0.06,  0.01] |   -1.65 | 0.099 
ilr factor [2] × age                               | [-0.03,  0.10] |    0.99 | 0.324 
ilr factor [3] × age                               | [-0.02,  0.04] |    0.57 | 0.570 
ilr factor [1] × sexFemale                         | [ 0.00,  0.12] |    2.02 | 0.044 
ilr factor [2] × sexFemale                         | [-0.24, -0.03] |   -2.53 | 0.011 
ilr factor [3] × sexFemale                         | [ 0.04,  0.15] |    3.53 | < .001
ilr factor [1] × familySingle parent               | [-0.05,  0.07] |    0.23 | 0.816 
ilr factor [2] × familySingle parent               | [-0.08,  0.13] |    0.39 | 0.699 
ilr factor [3] × familySingle parent               | [-0.11,  0.01] |   -1.59 | 0.112 
ilr factor [1] × familyDoest not live with parents | [-0.08,  0.18] |    0.78 | 0.438 
ilr factor [2] × familyDoest not live with parents | [-0.32,  0.11] |   -0.97 | 0.335 
ilr factor [3] × familyDoest not live with parents | [-0.11,  0.14] |    0.25 | 0.801 
ilr factor [1] × ses                               | [ 0.00,  0.00] |    0.64 | 0.523 
ilr factor [2] × ses                               | [ 0.00,  0.01] |    0.39 | 0.700 
ilr factor [3] × ses                               | [ 0.00,  0.00] |   -0.73 | 0.465 

# Random Effects

Parameter                                | Coefficient
------------------------------------------------------
SD (ilr_factor1: id:campus)              |        0.16
SD (ilr_factor2: id:campus)              |        0.38
SD (ilr_factor3: id:campus)              |        0.14
SD (ilr_factor1: campus)                 |        0.05
SD (ilr_factor2: campus)                 |        0.09
SD (ilr_factor3: campus)                 |        0.05
Cor (ilr_factor1~ilr_factor2: id:campus) |       -0.97
Cor (ilr_factor1~ilr_factor3: id:campus) |       -0.77
Cor (ilr_factor2~ilr_factor3: id:campus) |        0.59
Cor (ilr_factor1~ilr_factor2: campus)    |       -1.00
Cor (ilr_factor1~ilr_factor3: campus)    |       -0.52
Cor (ilr_factor2~ilr_factor3: campus)    |        0.52
SD (Residual)                            |        0.19
```

```
# Combining parameters in a single object

coef <- bind_rows(c1_ilr_mvpa, c1_ilr_lipa, c1_ilr_sb, c1_ilr_spt) %>% 
  mutate(parameter = labels) %>% 
  bind_rows(comp) 

coef <- coef[c(5,1,2,3,4), ]
```

## 2.5 Summary (Table 4)

Parameters of interest from the models

```
tbl4 <- bind_cols(pred, coef) %>% 
  mutate(
    p = as.character(p),
    p = case_match(
      p,
      '0.000' ~ '<0.001',
      .default = p),
    change =  sprintf((`2022`/`2019`-1)*100, fmt = "%.1f")) %>% 
  relocate(parameter, `2019`, `2022`, change) 

tbl4 %>% 
  kbl(align = 'c',
      col.names = c('Outcomes', '2019 Pred.avg', '2022 Pred.avg', 'Difference (%)', 'Coef', 'p')) %>% 
  kable_styling(bootstrap_options = c("striped", "hover", "condensed", "responsive"))
```

| Outcomes | 2019 Pred.avg | 2022 Pred.avg | Difference (%) | Coef | p |
| --- | --- | --- | --- | --- | --- |
| Composition | NA | NA | NA | NA | <0.001 |
| MVPA | 30.5 | 25.1 | -17.7 | -0.160 | <0.001 |
| LIPA | 277.4 | 261.1 | -5.9 | -0.002 | 0.923 |
| SB | 671.5 | 705.5 | 5.1 | 0.125 | <0.001 |
| SPT | 460.6 | 448.4 | -2.6 | 0.037 | 0.084 |

## 2.6 Moderation analysis

The third set of models were fitted to test whether the effect of time point on the composition varied according to sociodemographic characteristics.

```
moderators <- c('sex', 'age', 'family', 'ses')

for (moderator in moderators) {
  
  formula <- paste0('ilr ~ -1 +
                  ilr_factor +
                  ilr_factor:survey +
                  ilr_factor:age +
                  ilr_factor:sex +
                  ilr_factor:family +
                  ilr_factor:ses +
                  ilr_factor:survey:', 
                  moderator,
                  ' + (0 + ilr_factor | campus/id)')
  options(contrasts = c('contr.sum','contr.poly'))
  model <- lmer(formula, 
                coda_stacked,
                control = lmerControl(
                  optimizer ='optimx', optCtrl=list(method='L-BFGS-B'),
                  check.conv.singular = 
                    .makeCC(action = "ignore", 
                            tol = formals(isSingular)$tol)))
  print(' ')
  print(moderator)
  parameters(model) %>% print()
  
  parameters(car::Anova(model, type = "III")) %>% 
    print()
  
  effect <- parameters(car::Anova(model, type = "III")) %>% 
    filter(Parameter == paste0('ilr_factor:survey:', moderator)) %>% 
    select(Parameter, df, Chi2, p) %>% 
    mutate(
      Parameter = paste0('ilr_factor * survey * ', moderator),
      p = sprintf(p, fmt = "%.3f")) %>% 
    clean_names() %>% 
    as_tibble()
  
  assign(paste0('inter_', moderator), effect)
  remove(list = c('effect'))
}
```

```
[1] " "
[1] "sex"
# Fixed Effects

Parameter                       | Coefficient |       SE |         95% CI
-------------------------------------------------------------------------
ilr factor [1]                  |        2.04 |     0.43 | [ 1.19,  2.90]
ilr factor [2]                  |        2.08 |     0.42 | [ 1.25,  2.90]
ilr factor [3]                  |        1.26 |     0.29 | [ 0.69,  1.83]
ilr factor [1] × survey1        |       -0.05 |     0.01 | [-0.07, -0.03]
ilr factor [2] × survey1        |       -0.07 |     0.01 | [-0.09, -0.05]
ilr factor [3] × survey1        |       -0.02 |     0.01 | [-0.04,  0.00]
ilr factor [1] × age            |       -0.02 |     0.03 | [-0.08,  0.03]
ilr factor [2] × age            |       -0.02 |     0.03 | [-0.08,  0.03]
ilr factor [3] × age            |       -0.03 |     0.02 | [-0.06,  0.01]
ilr factor [1] × sex1           |       -0.09 |     0.02 | [-0.13, -0.04]
ilr factor [2] × sex1           |    7.90e-03 |     0.02 | [-0.03,  0.05]
ilr factor [3] × sex1           |       -0.03 |     0.01 | [-0.06,  0.00]
ilr factor [1] × family1        |       -0.02 |     0.04 | [-0.09,  0.05]
ilr factor [2] × family1        |       -0.02 |     0.04 | [-0.09,  0.05]
ilr factor [3] × family1        |       -0.02 |     0.03 | [-0.07,  0.03]
ilr factor [1] × family2        |       -0.06 |     0.04 | [-0.14,  0.01]
ilr factor [2] × family2        |    6.65e-03 |     0.04 | [-0.06,  0.08]
ilr factor [3] × family2        |       -0.01 |     0.03 | [-0.06,  0.04]
ilr factor [1] × ses            |   -1.47e-03 | 2.36e-03 | [-0.01,  0.00]
ilr factor [2] × ses            |    3.81e-04 | 2.28e-03 | [ 0.00,  0.00]
ilr factor [3] × ses            |    1.06e-03 | 1.57e-03 | [ 0.00,  0.00]
ilr factor [1] × survey1 × sex1 |        0.01 |     0.01 | [-0.01,  0.03]
ilr factor [2] × survey1 × sex1 |   -1.77e-04 |     0.01 | [-0.02,  0.02]
ilr factor [3] × survey1 × sex1 |        0.01 |     0.01 | [-0.01,  0.03]

Parameter                       | t(1124) |      p
--------------------------------------------------
ilr factor [1]                  |    4.71 | < .001
ilr factor [2]                  |    4.95 | < .001
ilr factor [3]                  |    4.31 | < .001
ilr factor [1] × survey1        |   -4.24 | < .001
ilr factor [2] × survey1        |   -6.56 | < .001
ilr factor [3] × survey1        |   -1.65 | 0.098 
ilr factor [1] × age            |   -0.87 | 0.385 
ilr factor [2] × age            |   -0.96 | 0.335 
ilr factor [3] × age            |   -1.62 | 0.106 
ilr factor [1] × sex1           |   -3.96 | < .001
ilr factor [2] × sex1           |    0.37 | 0.714 
ilr factor [3] × sex1           |   -2.26 | 0.024 
ilr factor [1] × family1        |   -0.51 | 0.613 
ilr factor [2] × family1        |   -0.65 | 0.517 
ilr factor [3] × family1        |   -0.72 | 0.471 
ilr factor [1] × family2        |   -1.61 | 0.107 
ilr factor [2] × family2        |    0.18 | 0.855 
ilr factor [3] × family2        |   -0.44 | 0.658 
ilr factor [1] × ses            |   -0.62 | 0.534 
ilr factor [2] × ses            |    0.17 | 0.867 
ilr factor [3] × ses            |    0.67 | 0.500 
ilr factor [1] × survey1 × sex1 |    1.05 | 0.292 
ilr factor [2] × survey1 × sex1 |   -0.02 | 0.987 
ilr factor [3] × survey1 × sex1 |    1.17 | 0.242 

# Random Effects

Parameter                                | Coefficient
------------------------------------------------------
SD (ilr_factor1: id:campus)              |        0.29
SD (ilr_factor2: id:campus)              |        0.28
SD (ilr_factor3: id:campus)              |        0.16
SD (ilr_factor1: campus)                 |        0.07
SD (ilr_factor2: campus)                 |        0.07
SD (ilr_factor3: campus)                 |        0.05
Cor (ilr_factor1~ilr_factor2: id:campus) |        0.85
Cor (ilr_factor1~ilr_factor3: id:campus) |        0.90
Cor (ilr_factor2~ilr_factor3: id:campus) |        0.99
Cor (ilr_factor1~ilr_factor2: campus)    |        0.69
Cor (ilr_factor1~ilr_factor3: campus)    |        0.93
Cor (ilr_factor2~ilr_factor3: campus)    |        0.91
SD (Residual)                            |        0.20
```

```
Parameter             |  Chi2 | df |      p
-------------------------------------------
ilr_factor            | 29.85 |  3 | < .001
ilr_factor:survey     | 59.32 |  3 | < .001
ilr_factor:age        |  2.62 |  3 | 0.454 
ilr_factor:sex        | 32.35 |  3 | < .001
ilr_factor:family     |  5.40 |  6 | 0.493 
ilr_factor:ses        |  1.99 |  3 | 0.574 
ilr_factor:survey:sex |  2.40 |  3 | 0.494 

Anova Table (Type 3 tests)
```

```
[1] " "
[1] "age"
# Fixed Effects

Parameter                      | Coefficient |       SE |         95% CI
------------------------------------------------------------------------
ilr factor [1]                 |        1.95 |     0.45 | [ 1.06,  2.84]
ilr factor [2]                 |        2.25 |     0.44 | [ 1.39,  3.11]
ilr factor [3]                 |        1.36 |     0.31 | [ 0.74,  1.97]
ilr factor [1] × survey1       |        0.17 |     0.24 | [-0.30,  0.64]
ilr factor [2] × survey1       |       -0.39 |     0.24 | [-0.86,  0.07]
ilr factor [3] × survey1       |       -0.19 |     0.22 | [-0.62,  0.25]
ilr factor [1] × age           |       -0.02 |     0.03 | [-0.07,  0.04]
ilr factor [2] × age           |       -0.04 |     0.03 | [-0.09,  0.02]
ilr factor [3] × age           |       -0.04 |     0.02 | [-0.07,  0.00]
ilr factor [1] × sex1          |       -0.08 |     0.02 | [-0.13, -0.04]
ilr factor [2] × sex1          |    8.81e-03 |     0.02 | [-0.03,  0.05]
ilr factor [3] × sex1          |       -0.03 |     0.01 | [-0.06,  0.00]
ilr factor [1] × family1       |       -0.02 |     0.04 | [-0.09,  0.05]
ilr factor [2] × family1       |       -0.02 |     0.04 | [-0.09,  0.05]
ilr factor [3] × family1       |       -0.02 |     0.03 | [-0.07,  0.03]
ilr factor [1] × family2       |       -0.06 |     0.04 | [-0.14,  0.01]
ilr factor [2] × family2       |    8.46e-03 |     0.04 | [-0.06,  0.08]
ilr factor [3] × family2       |       -0.01 |     0.03 | [-0.06,  0.04]
ilr factor [1] × ses           |   -1.47e-03 | 2.36e-03 | [-0.01,  0.00]
ilr factor [2] × ses           |    3.09e-04 | 2.28e-03 | [ 0.00,  0.00]
ilr factor [3] × ses           |    9.60e-04 | 1.57e-03 | [ 0.00,  0.00]
ilr factor [1] × survey1 × age |       -0.01 |     0.02 | [-0.04,  0.02]
ilr factor [2] × survey1 × age |        0.02 |     0.02 | [-0.01,  0.05]
ilr factor [3] × survey1 × age |        0.01 |     0.01 | [-0.02,  0.04]

Parameter                      | t(1124) |      p
-------------------------------------------------
ilr factor [1]                 |    4.30 | < .001
ilr factor [2]                 |    5.12 | < .001
ilr factor [3]                 |    4.34 | < .001
ilr factor [1] × survey1       |    0.70 | 0.482 
ilr factor [2] × survey1       |   -1.65 | 0.099 
ilr factor [3] × survey1       |   -0.83 | 0.408 
ilr factor [1] × age           |   -0.60 | 0.547 
ilr factor [2] × age           |   -1.33 | 0.184 
ilr factor [3] × age           |   -1.82 | 0.069 
ilr factor [1] × sex1          |   -3.81 | < .001
ilr factor [2] × sex1          |    0.42 | 0.677 
ilr factor [3] × sex1          |   -2.03 | 0.043 
ilr factor [1] × family1       |   -0.54 | 0.587 
ilr factor [2] × family1       |   -0.62 | 0.539 
ilr factor [3] × family1       |   -0.72 | 0.472 
ilr factor [1] × family2       |   -1.62 | 0.105 
ilr factor [2] × family2       |    0.23 | 0.817 
ilr factor [3] × family2       |   -0.44 | 0.663 
ilr factor [1] × ses           |   -0.62 | 0.534 
ilr factor [2] × ses           |    0.14 | 0.892 
ilr factor [3] × ses           |    0.61 | 0.541 
ilr factor [1] × survey1 × age |   -0.90 | 0.367 
ilr factor [2] × survey1 × age |    1.35 | 0.178 
ilr factor [3] × survey1 × age |    0.75 | 0.456 

# Random Effects

Parameter                                | Coefficient
------------------------------------------------------
SD (ilr_factor1: id:campus)              |        0.29
SD (ilr_factor2: id:campus)              |        0.28
SD (ilr_factor3: id:campus)              |        0.16
SD (ilr_factor1: campus)                 |        0.07
SD (ilr_factor2: campus)                 |        0.07
SD (ilr_factor3: campus)                 |        0.05
Cor (ilr_factor1~ilr_factor2: id:campus) |        0.85
Cor (ilr_factor1~ilr_factor3: id:campus) |        0.91
Cor (ilr_factor2~ilr_factor3: id:campus) |        0.99
Cor (ilr_factor1~ilr_factor2: campus)    |        0.68
Cor (ilr_factor1~ilr_factor3: campus)    |        0.92
Cor (ilr_factor2~ilr_factor3: campus)    |        0.91
SD (Residual)                            |        0.20
```

```
Parameter             |  Chi2 | df |      p
-------------------------------------------
ilr_factor            | 30.45 |  3 | < .001
ilr_factor:survey     |  3.98 |  3 | 0.264 
ilr_factor:age        |  3.81 |  3 | 0.282 
ilr_factor:sex        | 31.23 |  3 | < .001
ilr_factor:family     |  5.54 |  6 | 0.477 
ilr_factor:ses        |  1.82 |  3 | 0.611 
ilr_factor:survey:age |  3.33 |  3 | 0.343 

Anova Table (Type 3 tests)
```

```
[1] " "
[1] "family"
# Fixed Effects

Parameter                          | Coefficient |       SE |         95% CI
----------------------------------------------------------------------------
ilr factor [1]                     |        2.06 |     0.43 | [ 1.21,  2.91]
ilr factor [2]                     |        2.08 |     0.42 | [ 1.25,  2.90]
ilr factor [3]                     |        1.27 |     0.29 | [ 0.70,  1.85]
ilr factor [1] × survey1           |       -0.03 |     0.02 | [-0.07,  0.01]
ilr factor [2] × survey1           |       -0.09 |     0.02 | [-0.12, -0.05]
ilr factor [3] × survey1           |       -0.01 |     0.02 | [-0.05,  0.02]
ilr factor [1] × age               |       -0.02 |     0.03 | [-0.08,  0.03]
ilr factor [2] × age               |       -0.02 |     0.03 | [-0.07,  0.03]
ilr factor [3] × age               |       -0.03 |     0.02 | [-0.06,  0.01]
ilr factor [1] × sex1              |       -0.08 |     0.02 | [-0.13, -0.04]
ilr factor [2] × sex1              |    9.27e-03 |     0.02 | [-0.03,  0.05]
ilr factor [3] × sex1              |       -0.03 |     0.01 | [-0.06,  0.00]
ilr factor [1] × family1           |       -0.02 |     0.04 | [-0.09,  0.05]
ilr factor [2] × family1           |       -0.03 |     0.04 | [-0.10,  0.04]
ilr factor [3] × family1           |       -0.02 |     0.03 | [-0.07,  0.03]
ilr factor [1] × family2           |       -0.05 |     0.04 | [-0.13,  0.02]
ilr factor [2] × family2           |    2.06e-03 |     0.04 | [-0.07,  0.07]
ilr factor [3] × family2           |   -9.96e-03 |     0.03 | [-0.06,  0.04]
ilr factor [1] × ses               |   -1.51e-03 | 2.36e-03 | [-0.01,  0.00]
ilr factor [2] × ses               |    3.82e-04 | 2.28e-03 | [ 0.00,  0.00]
ilr factor [3] × ses               |    1.00e-03 | 1.57e-03 | [ 0.00,  0.00]
ilr factor [1] × survey1 × family1 |   -6.51e-03 |     0.02 | [-0.05,  0.04]
ilr factor [2] × survey1 × family1 |        0.01 |     0.02 | [-0.03,  0.05]
ilr factor [3] × survey1 × family1 |   -1.91e-03 |     0.02 | [-0.04,  0.04]
ilr factor [1] × survey1 × family2 |       -0.04 |     0.02 | [-0.09,  0.00]
ilr factor [2] × survey1 × family2 |        0.02 |     0.02 | [-0.02,  0.07]
ilr factor [3] × survey1 × family2 |       -0.01 |     0.02 | [-0.05,  0.03]

Parameter                          | t(1121) |      p
-----------------------------------------------------
ilr factor [1]                     |    4.76 | < .001
ilr factor [2]                     |    4.94 | < .001
ilr factor [3]                     |    4.34 | < .001
ilr factor [1] × survey1           |   -1.65 | 0.100 
ilr factor [2] × survey1           |   -4.47 | < .001
ilr factor [3] × survey1           |   -0.77 | 0.443 
ilr factor [1] × age               |   -0.90 | 0.366 
ilr factor [2] × age               |   -0.95 | 0.340 
ilr factor [3] × age               |   -1.65 | 0.100 
ilr factor [1] × sex1              |   -3.86 | < .001
ilr factor [2] × sex1              |    0.44 | 0.662 
ilr factor [3] × sex1              |   -2.04 | 0.041 
ilr factor [1] × family1           |   -0.59 | 0.557 
ilr factor [2] × family1           |   -0.74 | 0.462 
ilr factor [3] × family1           |   -0.77 | 0.440 
ilr factor [1] × family2           |   -1.34 | 0.179 
ilr factor [2] × family2           |    0.06 | 0.955 
ilr factor [3] × family2           |   -0.37 | 0.710 
ilr factor [1] × ses               |   -0.64 | 0.522 
ilr factor [2] × ses               |    0.17 | 0.867 
ilr factor [3] × ses               |    0.64 | 0.523 
ilr factor [1] × survey1 × family1 |   -0.31 | 0.759 
ilr factor [2] × survey1 × family1 |    0.62 | 0.533 
ilr factor [3] × survey1 × family1 |   -0.10 | 0.923 
ilr factor [1] × survey1 × family2 |   -1.92 | 0.055 
ilr factor [2] × survey1 × family2 |    0.95 | 0.343 
ilr factor [3] × survey1 × family2 |   -0.55 | 0.579 

# Random Effects

Parameter                                | Coefficient
------------------------------------------------------
SD (ilr_factor1: id:campus)              |        0.29
SD (ilr_factor2: id:campus)              |        0.28
SD (ilr_factor3: id:campus)              |        0.16
SD (ilr_factor1: campus)                 |        0.07
SD (ilr_factor2: campus)                 |        0.07
SD (ilr_factor3: campus)                 |        0.05
Cor (ilr_factor1~ilr_factor2: id:campus) |        0.85
Cor (ilr_factor1~ilr_factor3: id:campus) |        0.90
Cor (ilr_factor2~ilr_factor3: id:campus) |        0.99
Cor (ilr_factor1~ilr_factor2: campus)    |        0.71
Cor (ilr_factor1~ilr_factor3: campus)    |        0.93
Cor (ilr_factor2~ilr_factor3: campus)    |        0.91
SD (Residual)                            |        0.20
```

```
Parameter                |  Chi2 | df |      p
----------------------------------------------
ilr_factor               | 30.13 |  3 | < .001
ilr_factor:survey        | 21.68 |  3 | < .001
ilr_factor:age           |  2.72 |  3 | 0.437 
ilr_factor:sex           | 32.12 |  3 | < .001
ilr_factor:family        |  3.66 |  6 | 0.723 
ilr_factor:ses           |  1.96 |  3 | 0.580 
ilr_factor:survey:family |  5.52 |  6 | 0.479 

Anova Table (Type 3 tests)
```

```
[1] " "
[1] "ses"
# Fixed Effects

Parameter                      | Coefficient |       SE |         95% CI
------------------------------------------------------------------------
ilr factor [1]                 |        2.03 |     0.43 | [ 1.18,  2.89]
ilr factor [2]                 |        2.04 |     0.42 | [ 1.22,  2.87]
ilr factor [3]                 |        1.25 |     0.29 | [ 0.68,  1.83]
ilr factor [1] × survey1       |   -9.63e-03 |     0.05 | [-0.10,  0.08]
ilr factor [2] × survey1       |    6.00e-03 |     0.04 | [-0.08,  0.09]
ilr factor [3] × survey1       |        0.02 |     0.04 | [-0.07,  0.10]
ilr factor [1] × age           |       -0.02 |     0.03 | [-0.08,  0.03]
ilr factor [2] × age           |       -0.02 |     0.03 | [-0.07,  0.03]
ilr factor [3] × age           |       -0.03 |     0.02 | [-0.06,  0.01]
ilr factor [1] × sex1          |       -0.08 |     0.02 | [-0.13, -0.04]
ilr factor [2] × sex1          |    7.61e-03 |     0.02 | [-0.03,  0.05]
ilr factor [3] × sex1          |       -0.03 |     0.01 | [-0.06,  0.00]
ilr factor [1] × family1       |       -0.02 |     0.04 | [-0.09,  0.05]
ilr factor [2] × family1       |       -0.02 |     0.04 | [-0.09,  0.05]
ilr factor [3] × family1       |       -0.02 |     0.03 | [-0.07,  0.03]
ilr factor [1] × family2       |       -0.06 |     0.04 | [-0.14,  0.01]
ilr factor [2] × family2       |    4.56e-03 |     0.04 | [-0.07,  0.08]
ilr factor [3] × family2       |       -0.01 |     0.03 | [-0.07,  0.04]
ilr factor [1] × ses           |   -1.15e-03 | 2.39e-03 | [-0.01,  0.00]
ilr factor [2] × ses           |    1.07e-03 | 2.30e-03 | [ 0.00,  0.01]
ilr factor [3] × ses           |    1.32e-03 | 1.60e-03 | [ 0.00,  0.00]
ilr factor [1] × survey1 × ses |   -1.00e-03 | 1.14e-03 | [ 0.00,  0.00]
ilr factor [2] × survey1 × ses |   -2.04e-03 | 1.13e-03 | [ 0.00,  0.00]
ilr factor [3] × survey1 × ses |   -9.09e-04 | 1.09e-03 | [ 0.00,  0.00]

Parameter                      | t(1124) |      p
-------------------------------------------------
ilr factor [1]                 |    4.68 | < .001
ilr factor [2]                 |    4.86 | < .001
ilr factor [3]                 |    4.28 | < .001
ilr factor [1] × survey1       |   -0.21 | 0.831 
ilr factor [2] × survey1       |    0.13 | 0.894 
ilr factor [3] × survey1       |    0.38 | 0.701 
ilr factor [1] × age           |   -0.87 | 0.383 
ilr factor [2] × age           |   -0.95 | 0.344 
ilr factor [3] × age           |   -1.62 | 0.105 
ilr factor [1] × sex1          |   -3.83 | < .001
ilr factor [2] × sex1          |    0.36 | 0.719 
ilr factor [3] × sex1          |   -2.07 | 0.039 
ilr factor [1] × family1       |   -0.49 | 0.625 
ilr factor [2] × family1       |   -0.62 | 0.538 
ilr factor [3] × family1       |   -0.72 | 0.473 
ilr factor [1] × family2       |   -1.68 | 0.093 
ilr factor [2] × family2       |    0.13 | 0.901 
ilr factor [3] × family2       |   -0.52 | 0.605 
ilr factor [1] × ses           |   -0.48 | 0.632 
ilr factor [2] × ses           |    0.46 | 0.643 
ilr factor [3] × ses           |    0.83 | 0.409 
ilr factor [1] × survey1 × ses |   -0.88 | 0.379 
ilr factor [2] × survey1 × ses |   -1.81 | 0.071 
ilr factor [3] × survey1 × ses |   -0.84 | 0.403 

# Random Effects

Parameter                                | Coefficient
------------------------------------------------------
SD (ilr_factor1: id:campus)              |        0.30
SD (ilr_factor2: id:campus)              |        0.28
SD (ilr_factor3: id:campus)              |        0.16
SD (ilr_factor1: campus)                 |        0.07
SD (ilr_factor2: campus)                 |        0.07
SD (ilr_factor3: campus)                 |        0.05
Cor (ilr_factor1~ilr_factor2: id:campus) |        0.85
Cor (ilr_factor1~ilr_factor3: id:campus) |        0.90
Cor (ilr_factor2~ilr_factor3: id:campus) |        0.99
Cor (ilr_factor1~ilr_factor2: campus)    |        0.69
Cor (ilr_factor1~ilr_factor3: campus)    |        0.93
Cor (ilr_factor2~ilr_factor3: campus)    |        0.91
SD (Residual)                            |        0.19
```

```
Parameter             |  Chi2 | df |      p
-------------------------------------------
ilr_factor            | 29.18 |  3 | < .001
ilr_factor:survey     |  0.22 |  3 | 0.975 
ilr_factor:age        |  2.64 |  3 | 0.450 
ilr_factor:sex        | 30.85 |  3 | < .001
ilr_factor:family     |  5.52 |  6 | 0.479 
ilr_factor:ses        |  2.22 |  3 | 0.529 
ilr_factor:survey:ses |  4.40 |  3 | 0.222 

Anova Table (Type 3 tests)
```

## 2.7 Summary (Table 5.2)

Parameters of interest from the models

```
tbl52 <- bind_rows(inter_sex, inter_age, inter_family, inter_ses) %>% 
  mutate(
    p = as.character(p),
    p = case_match(
      p,
      '0.000' ~ '<0.001',
      .default = p))

tbl52 %>% 
  kbl(align = 'l',
      caption = 'Interaction effect between each moderator and survey for the composition') %>% 
  kable_styling(bootstrap_options = c("striped", "hover", "condensed", "responsive"))
```

Interaction effect between each moderator and survey for the composition

| parameter | df | chi2 | p |
| --- | --- | --- | --- |
| ilr\_factor \* survey \* sex | 3 | 2.399770 | 0.494 |
| ilr\_factor \* survey \* age | 3 | 3.333978 | 0.343 |
| ilr\_factor \* survey \* family | 6 | 5.517600 | 0.479 |
| ilr\_factor \* survey \* ses | 3 | 4.397752 | 0.222 |

## References

Lim, Renly, Dorothea Dumuid, Gaynor Parfitt, Tyson Stanford, Dannielle Post, Rebecca Bilton, Lisa M. Kalisch Ellett, Nicole Pratt, and Elizabeth E. Roughead. 2023. “Using Wrist-Worn Accelerometers to Identify the Impact of Medicines with Anticholinergic or Sedative Properties on Sedentary Time: A 12-Month Prospective Analysis.” *Maturitas* 172 (June): 9–14. https://doi.org/10.1016/j.maturitas.2023.03.006.

Rosen, Philip von. 2023. “Analysing Time-Use Composition as Dependent Variables in Physical Activity and Sedentary Behaviour Research: Different Compositional Data Analysis Approaches.” *Journal of Activity, Sedentary and Sleep Behaviors* 2 (1): 23. https://doi.org/10.1186/s44167-023-00033-5.
