# Supplementary material for: Assessing the damage: analyzing the impact of the COVID-19 pandemic on accelerometer-assessed 24-hour movement behaviours in Brazilian adolescents
Source: BMC Public Health. 2025 May 31;25:2022. doi: 10.1186/s12889-025-23155-8 (PMC12125940; doi:10.1186/s12889-025-23155-8)
Supplement: Supplementary file 3 — Additional file 3 contains the additional findings related with the sensitivity analysis. [file 12889_2025_23155_MOESM3_ESM.pdf]

Additional File 3

Marcus Lopes (mveber@cheo.on.ca)

Sensitivity analyses related to the article 'Assessing the damage: analyzing the impact of the COVID-19 pandemic on accelerometer-assessed 24-hour movement behaviours in Brazilian adolescents' authored by Marcus V V Lopes, Ian Janssen, Bruno G G da Costa, Bruno N de Oliveira, Gabrielli T de Mello, Jean-Philippe Chaput, Kelly S Silva

Table S1. Comparison of 24-hour movement behaviour compositions between 2019 and 2022 cross-sectional samples using adult cut-off points for behaviour classification.

Outcomes	Adjusted predictions (min/day) [a]		Difference (%)	p-value
	2019 sample	2022 sample		
Composition				0.061
MVPA	105.4	100.5	-4.6	0.001
LIPA	148.8	148.5	-0.2	0.411
SB	732.2	736.8	0.6	0.094
SPT	453.6	454.2	0.1	0.242

Note: Models were adjusted for age, sex, SES and family structure. [a] Marginal means adjusted for mean age and proportionally weighted according to the levels of factor covariates. MVPA, moderate-to-vigorous-intensity physical activity; LIPA, light-intensity physical activity; SB, sedentary behaviour; SPT, sleep duration.

Table S2. Within-participant changes in 24-hour movement behaviour compositions between 2019 and 2022 using adult cut-off points for behaviour classification.

Outcomes	Adjusted predictions (min/day) [a]		Difference (%)	p-value
	2019 sample	2022 sample		
Composition				<0.001
MVPA	108	95.1	-11.9	<0.001
LIPA	148.9	140.3	-5.8	0.232
SB	723.3	756.8	4.6	<0.001
SPT	459.7	447.8	-2.6	0.277

Note: Models were adjusted for sociodemographic characteristics at 2019 (i.e., age, sex, SES and family structure). [a] Marginal means adjusted for mean age and proportionally weighted according to the levels of factor covariates. MVPA, moderate-to-vigorous-intensity physical activity; LIPA, light-intensity physical activity; SB, sedentary behaviour; SPT, sleep duration.

Table S3. Moderation effects of sociodemographic factors on the comparisons of movement composition between 2019 and 2022 using adult cut-off points for behaviour classification.

Interaction terms	df	Chi	p-value*
**Cross-sectional sample**			
ILR * Survey * Sex	3	0.57272	0.903
ILR * Survey * Age	3	1.75943	0.624
ILR * Survey * Family structure	6	4.98154	0.546
ILR * Survey * SES	3	1.2148	0.749
**Longitudinal sample**			
ILR * Survey * Sex	3	3.46649	0.325
ILR * Survey * Age	3	4.15385	0.245
ILR * Survey * Family structure	6	5.40352	0.493
ILR * Survey * SES	3	4.58498	0.205

Note: * Wald test; ILR, Isometric log-ratio, SES, Socioeconomic status
